# NOMEN — A classification of methods used to create odourous compositions with a historical dimension: re-weighing, adapting, reconstructing, transposing, evoking

**DOI:** 10.12688/openreseurope.21121.1

**Published:** 2025-12-10

**Authors:** Isabelle Chazot, Érika Wicky, Sophie-Valentine Borloz, Chantal Jaquet, Alice Camus, Olivier David

**Affiliations:** 1 Laboratoire de Recherche Historique Rhône-Alpes, Lyon, Auvergne-Rhône-Alpes, France; 2 Université Grenoble Alpes UFR Arts et sciences humaines, Saint-Martin-d'Hères, Auvergne-Rhône-Alpes, France; 3 Independant researcher, Lausanne, Switzerland; 4 Université Paris 1 Panthéon-Sorbonne UFR 10 Philosophie, Paris, Île-de-France, France; 5 Independent researcher, Paris, France; 6 Institut Lavoisier de Versailles, Versailles, Île-de-France, France; 7 Université de Versailles Saint-Quentin-en-Yvelines, Versailles, Île-de-France, France

**Keywords:** Olfactory heritage, Olfactory reconstruction, scent of the past, sensory museology, historical perfumes, historical reconstitution, olfactory reconstitution, perfume of the past, classification of odorous compositions, experimental history

## Abstract

The past decade has seen a proliferation of olfactory compositions and devices claiming a degree of mimetic connection to a vanished scent or atmosphere, whether in museum settings or the context of scientific, commercial, educational, or event-based initiatives. The aim is to offer visitors a direct sensory connection to the past. These endeavours differ significantly in both their objectives and methodologies as they may include to re-create a lost perfume for which we have the original formula, reconstruct an ancient fragrance through archaeological analysis, or draw inspiration from literary or visual sources to evoke the scent of a particular place or time. The specific objectives of a given project, such as commercial purposes, also shape the perfumer’s brief. Despite their obvious differences, all such projects are often lumped together under the broad and vague label of “reconstitution,” with little or no indication of the methodology used or the scientific grounding of the work. This ambiguity undermines the historical credibility of the scents and tends to blur the scientific rigour behind its creation, the expertise of the perfumer who formulated it, and the historical research that informed both.

It is therefore essential to adopt a more nuanced terminology. With this aim in mind, and with the goal of mapping a highly dynamic and evolving field in which it has long played both an active and observer role, the Osmothèque, Conservatoire International des Parfums, tasked its multidisciplinary scientific committee with developing a nomenclature to document these various odourous compositions and their specific characteristics. This classification was to be based on objective criteria and was independently conceived, without any evaluative or promotional intent. The result is the NOMEN model: a bilingual framework with five categories that encompass most of the methods currently employed to create compositions with a historical dimension.

## Introduction

Scents of antiquity, atmospheres of Symbolist salons, 18th-century wig powder, or incense from Imperial China: the past decade has seen a proliferation of olfactory compositions and devices claiming a degree of mimetic connection to a vanished scent or atmosphere, whether in museum settings or the context of scientific, commercial, educational, or event-based initiatives ^2^. The aim is to offer visitors a direct sensory connection to the past; in other words, to collapse temporal distance, even if only momentarily, by allowing them to physically experience the air of a bygone era.

These initiatives have the obvious advantage of promoting olfactory education and culture among the public while also drawing attention to the sensory dimension of history by highlighting it within the broader perspectives both of the history of the senses and history through the senses. By engaging with the past through the sense of smell, they align closely with the immersive and experiential approaches that are becoming increasingly central to cultural and scientific mediation. At the same time, they focus attention on a rich but often overlooked olfactory heritage, and toward the expertise of the perfumers who preserve these scents or create compelling contemporary interpretations.

Despite these important points of convergence, such approaches may be based on divergent assumptions and methods, depending on the historical sources they are drawing upon, the period under consideration, and the techniques being employed. These endeavours may include to re-create a lost perfume for which we have the original formula, reconstruct an ancient fragrance through archaeological analysis, or draw inspiration from literary or visual sources to evoke the scent of a particular place or time. But, however fascinating the tasks are, they differ significantly in both their objectives and methodologies. Likewise, the specific objectives of a given project, such as commercial purposes, will inevitably shape the perfumer’s brief and influence which criteria are prioritised in the development of the scent.

And yet, despite their obvious differences, all such projects are often lumped together under the broad and vague label of “reconstitution,” with little or no indication of the methodology used or the scientific grounding of the work. Museums and exhibition spaces sometimes even bypass the process of olfactory reconstitution entirely, presenting scents labeled simply as “Kyphi” or “What you could smell in Versailles” without providing any information on the sources consulted or the techniques employed. This ambiguity undermines the historical credibility of the scents, as it is impossible to know exactly what is being smelled. It also obscures the uniqueness and value of each individual approach. Due to the vagueness of its designation, the term “reconstituted scent” (or sometimes “perfume”) tends to blur the scientific rigour behind its creation, the expertise of the perfumer who formulated it, and the historical research that informed both.

To avoid any misunderstanding regarding the status of these “reconstitutions”, ^3^ and to better recognise the expertise they involve, it is therefore essential to adopt a more nuanced terminology, that distinguishes between the various approaches and highlights their specific characteristics. While some ambiguity between categories is inevitable given that lost scents are, by nature, elusive and difficult to objectify, this effort at classification is a necessary first step toward clarifying current practices. Doing so not only benefits the general public, but also the perfumers and experts involved in these reconstructions, along with the museums and cultural institutions committed to providing accurate and informed experiences. It is important to emphasise that this proposed nomenclature is neither evaluative nor prescriptive; the goal is not to measure deviation from some “ideal” original scent. The availability of historical sources or the similarity of modern materials to ancient ones does not in itself determine the value of a given approach. Likewise, capturing the olfactory atmosphere of a historical period is a task that necessarily involves the creative interpretation of a perfumer.

Whether we speak of historical olfactory reconstitutions, odour-based compositions with historical resonance, or reimaginings of past scents, each of these approaches has genuine value. Audiences will be able to more meaningfully engage with them if they understand what kind of work is being presented. For this reason, we believe it is essential to develop a vocabulary that articulates the different ways of bringing lost scents back to life — and to raise awareness of the diversity of methods encompassed within the term “reconstitution.”

With this aim in mind, and with the goal of mapping a highly dynamic and evolving field in which it has long played both an active and observer role, the Osmothèque, Conservatoire International des Parfums, tasked its multidisciplinary scientific committee with developing a nomenclature to document these various odourous compositions and their specific characteristics. This classification was to be based on objective criteria and was independently conceived, without any evaluative or promotional intent. The result is the NOMEN model: a bilingual framework with five categories that encompass most of the methods currently employed to create compositions with a historical dimension.

The Osmothèque is a non-profit organization founded in France in 1990 under the French 1901 law on associations, which remains to this day the world’s only perfume conservatory and archive. Its mission is threefold: to preserve perfumes that have shaped (and continue to shape) the history of perfumery by recreating lost fragrances from original formulas; to share its collection, which now comprises over 6,000 perfumes, through public olfactory conferences; and to advance research on the history of perfume. As a pioneer in the preservation of olfactory heritage, the Osmothèque has taken a close interest in recent efforts to recreate historical scents.

### 1. State of the art

The NOMEN classification forms part of the critical approach to odorous compositions with a historical dimension, as the latter have become increasingly prevalent in cultural institutions. Despite their growing popularity, these compositions have received surprisingly little attention in the extensive literature on olfaction produced by scholars across multiple disciplines over the past decade. The fact that museology ( Curatola, 2020; Deramond & Pianezza, 2020; Levent & Pascual-Leone, 2014) is one of the few fields to have acknowledged the scope of this phenomenon may reflect the relative absence of historians from the actual process of creating historically-inspired scents. While historians are often involved upstream (e.g., in suggesting olfactory elements for the perfumer to emphasise) or downstream (e.g., in contributing to interpretive materials for public engagement), they remain largely removed from the creative phase itself, which is typically hidden within the private realm of the perfumer’s expertise and practice ( Marx *et al.*, 2022).

Yet, as was previously noted, the very nature of the composition, and its appropriateness for the intended project, depend closely on the method used to build the scent and how its raw materials were chosen. The nomenclature presented here seeks to address a gap in the current research by documenting the actual procedures involved in composing these fragrances. This approach is grounded in current scholarly work: methodologically, through the categorisation of practices and a critical reflection on their boundaries, a process which has often revealed the complexity and diversity of large creative fields, as, for example, Gérard Genette demonstrated in his typologies of artworks ( Genette, 1994). It also responds to the need expressed within the museum world to structure and conceptualise emerging immersive practices, as highlighted by the recent international symposium on “Museum Experience and New Immersive Devices” (France-Canada, October 10–11, 2024). Ultimately, the NOMEN classification emphasises that reconstituting past scents is far from a straightforward or monolithic process. Rather, it offers a framework for exploring the specific challenges and methodologies associated with each category in great depth, and in dialogue with a wide range of historiographical perspectives.

While the method referred to in the classification as *evocation* — a creative work inspired by the past (see below) is historically informed, it still allows significant room for the perfumer’s imagination and can therefore be appreciated aesthetically as a contemporary artistic creation. Conversely, the *Reweghing* ( *repesée)* approach, which aims to recreate original perfumes from the past as accurately as possible, opens the door to an aesthetic experience of a historical scent. The very notion that a perfume can constitute a work of art, and that smelling it can be an aesthetic experience, has long been debated, but recent research, notably by Chantal Jaquet ( 2010; 2018) and the team she assembled with Roland Salesse for the French National Research Agency’s Kôdô project ( Jaquet, 2015), has supported it. The artistic dimension of olfactory composition has also been explored in studies that have identified the emergence of a contemporary olfactory art form, with roots which can be traced back to the fin-de-siècle period ( Barré, 2021; Shiner, 2020). The classification proposed here serves as a bridge between historically oriented olfactory creations and the ongoing scholarly work on the intersection of art and scent.

Despite the creativity they require, historical olfactory reconstructions have rarely been considered from an aesthetic perspective, even though they can make meaningful contributions to ongoing debates between advocates of embodied sensory history ( Chen, 2016; Hoffer, 2003; Tullett, 2023) and critics, who argue that modern perception is too far removed from that of earlier societies to serve as a foundation for historical understanding ( Smith, 2007). Emily C. Friedman (2016), for instance, opens her book on literary representations of olfaction in the eighteenth century with reflections on her disappointingly unconvincing encounters with historical reconstructions.

While it is true that the evolution of our sensory frameworks limits direct access to past perceptions, experimental archaeology offers a compelling alternative. By replicating historical practices, it allows us to rediscover the techniques used by, and sensory engagement of, artisans and workers from earlier times, reconstruct production chains ( Dupré *et al.*, 2020), and reveal the role of the senses in craftsmanship ( Low & Abdullah, 2019; Sibum, 1995). A clear illustration of this is provided by the blog post documenting the reconstitution of a 17th-century perfume based on a recipe by the Dutch diplomat Constantijn Huygens (1596–1687). Conducted as part of the European DURARE project (2022), the experiment not only illuminated the distillation techniques used to produce hydrolats, but also explored the many variables that affected perfume-making in the period, including the freshness and type of the plants used, and the conditions under which the resulting product was stored.

*Reconstructions* (see below) engage directly with historical and archaeological research aimed at identifying the nature of the raw materials involved. They rely on the most advanced scientific and technical tools available ( Huber *et al.*, 2022) and require a deep understanding of the history of scent-making techniques, particularly those used in the past to extract and process raw materials. In this respect, methodological reflection on olfactory reconstruction can draw valuable insights from the practices of art restorers, who are adept at analysing materials and reproducing varnishes, waxes, glues, and paints to preserve the material authenticity of artworks ( Technè, 2008). These professionals also follow rigorous protocols to document their interventions and ensure the use of substances that closely match the original materials, both in composition and in compatibility, while adhering to strict safety and environmental protection requirements. This model of careful documentation and historically-grounded material reproduction offers a useful parallel for those working to reconstruct historical scents with scientific accuracy and integrity.

However, regardless of the historical insights which may emerge from the process of reconstituting a scent for historical purposes, they remain external to the scent itself. If these compositions carry historical significance, it is primarily through the processes they initiate rather than through the scent as an isolated object. The classification proposed here does not seek to evaluate the relevance of olfactory compositions as historical sources, but rather to describe and differentiate the various methods used to produce them so that they can be better understood, contextualised, and shared with the public. The aim of this classification is, therefore, also heritage-oriented in that it seeks to acknowledge and document the diverse approaches used to preserve the memory of meaningful scents from the past. By refining how we describe (and thus assess) the methods behind historically-inspired scent compositions, we are contributing to the broader preservation of olfactory heritage.

This attention to sensory heritage aligns with some current research, notably the work conducted by Cecilia Bembibre at the Institute for Sustainable Heritage (University College London). Her doctoral thesis, *Smell of Heritage* ( 2020), is a key milestone in this field ( Bembibre & Strilic, 2021; Bembibre & Strilic, 2022). Through a series of case studies, she examines in-depth issues such as the identification and archiving of reconstructed scents ( Bembibre & Strilic, 2017), and the evaluation of their perceived authenticity by the public. Building on this approach, Inger Leemans and the team behind the European *Odeuropa* project have produced several historical scent reconstitutions and developed large-scale data mining tools to explore digitised textual corpora, identifying historically significant odours, and in some cases recreating them for use in museums. Considered as narrative and representational tools, these historically-motivated reconstitutions are now the focus of growing efforts toward transparency. One such initiative is the *Whiffstory Toolkit*, which proposes a draft classification framework distinguishing between three types of historical sources (document-based, material- and knowledge-based, and artistic interpretation) and three types of olfactory targets (single ingredient, blended composition, and unpleasant odours) ( *Toolkit, Whiffstory).* ^4^

The research undertaken to develop the NOMEN classification is grounded in a joint analysis of the methodologies employed by historians (who document the scents to be reconstructed) and perfumers (who carry out the actual reconstitution process). In doing so, it addresses a significant gap in the scholarship on odour compositions with a historical dimension. At the same time, it aligns with, and contributes to, ongoing research by engaging with recent studies on the history of olfactory culture and the preservation of olfactory heritage.

### 2. Scope of application

Before presenting the different categories, a few preliminary remarks are necessary to clarify the scope and intent of the NOMEN model. First, this is not a classification of odours themselves, but a theoretical framework, which has been specifically designed to describe the methodologies used to create olfactory compositions that claim to have a historical dimension. By *historical dimension* in this context, we mean an explicit intention to recreate a lost scent that is known (or presumed) to have existed sometime in the past. Second, the classification is not limited to perfumes in the strict sense, ^5^ as it applies to all historical olfactory compositions in the broadest way. It is intended to encompass both efforts to recreate a specific perfume as faithfully as possible, and attempts to evoke the scent of a particular object, place, or environment from the past. Third, the model focuses on the methods, sources, and resources used in the creation process rather than on the olfactory outcome itself. It is therefore based on the most objective criteria available, avoiding any aesthetic or hedonic evaluation of the final product. Fourth and finally, the NOMEN classification is strictly descriptive; its purpose is to distinguish and highlight different types of creations, and not to judge or rank them. In short, NOMEN suggests a way to categorise any olfactory creation which has been brought into existence with the clear intention to reproduce a scent or perfume from the past, based on the information available about how it was produced.

The aim is to provide a useful tool for researchers, cultural institutions, and (through them) the wider public, while also enabling perfumers to define and communicate their own methodological approaches. By mapping the rapidly expanding field of historical olfactory reconstitutions, the classification opens up the diversity of expertise involved, underscores the methodological complexity of the work, and duly recognises the many contributors who make such reconstructions possible. ^6^

### 3. The NOMEN classification

Arranged along a continuum from the most complete and direct sources (offering the lowest degree of creative freedom) to the most indirect sources (allowing the greatest interpretive latitude) the NOMEN classification proposes five categories: *reweighing*, *adaptation*, *reconstruction*, *transposition*, and *evocation*. Each category is presented with illustrative examples below, not only to clarify the distinctions between them, but also to shed light on the specific challenges and underlying questions associated with each approach.

***Re-weighing***

***Re-weighing*** refers to cases in which the perfumer has access to a precise formula detailing the raw materials, proportions, and techniques which were originally used. This category concerns nominally identified perfumes, typically linked to a specific perfume house and perfumer-creator. Because it requires a formal and precise *formula* as opposed to a simple *recipe*, which tends to offer more approximate guidance (see “ *Adaptation*”), this approach generally applies to fragrances created from the late 19th century onward.

While perfumers had long recorded their work in recipe form, often combining lists of ingredients with references to tacit, empirical knowledge, the emergence of standardised formulas in the early 19th century (which were widely adopted from the 1880s onward) marked a turning point. These formulas are distinguished by their consistent structure and the precise documentation of both the ingredients and quantities. *Re-weighing* relies on the perfumer’s ability to remain as faithful as possible to the original formula, emphasising accuracy over creativity. Most of the perfumes recreated by the Osmothèque fall into this category of *re-weighing*.

However, having access to a perfume’s original formula is far from sufficient to complete a true *re-weighing*, as sourcing the specified raw materials can often be extremely challenging. For example, there is no guarantee whatsoever that a 2022 Centifolia rose absolute will smell exactly the same as one which was produced in 1910. While it would be unrealistic to recreate the precise natural environment in which the 1910 rose was grown, it is, however, possible to obtain the same species of flower and use the same extraction method, thereby minimising the variables as far as possible. ^7^ This example highlights the specific expertise required to evaluate and source raw materials, expertise that primarily lies with perfumers. Their deep knowledge of the history of perfumery allows them to infer, even when not explicitly stated in the formula, which type of rose absolute or essence was likely to have been used, and by which laboratory or perfumer, at a given time. Such professional insight is also crucial when dealing with rare or obsolete materials, whether they are of animal origin, discontinued natural extracts, or no longer produced synthetic compounds.

One of the major challenges in modern re-weighing relates to the use of *bases*. In perfumery, a base is a kind of “perfume core” — a pre-assembled accord sometimes designed to support a synthetic molecule (e.g., De Laire’s *Mousse de Saxe*), or to replicate a natural scent (e.g., Roure’s *Muguet 16*). These bases were commonly used as raw materials in their own right. The modern difficulty arises from the lack of available formulas for many of these bases; indeed, re-weighing perfumes that contain them can become impossible without that information. Fortunately, when archives have been preserved, some companies are able to re-weigh these bases and may donate small quantities to facilitate historical reconstructions. Collaborations of this sort, along with historical research (for instance, within industry archives such as the De Laire collection) have made it possible to recover valuable information about historical raw materials. Considering these obstacles, it is important to acknowledge that *re-weighing* can never result in a perfect reproduction of the original scent. ^8^ Moreover, perfumes — particularly those that rely heavily on natural ingredients — have always been “living” substances, which are inherently subject to climatic and biological variations. While the ideal of olfactory standardisation emerged from the technical and industrial advances of the 20th century, in practice it remains an aspiration, that can only ever be approached asymptotically.

Examples of true *re-weighings* are relatively rare. This is precisely the area of expertise of the Osmothèque — Conservatoire International des Parfums — which specialises in recreating perfumes as faithfully as possible to their original formulas, meticulously weighing each raw material with the highest possible precision. Since it was founded in 1990, the Conservatory has re-weighed approximately 300 perfumes using this method. Notable examples include *L'Origan* by Coty, re-weighed from an original 1905 formula, and *Le Fruit Défendu* by Parfums de Rosine (founded by Paul Poiret), re-weighed from its original 1914 formula. These perfumes are not intended for commercial sale; rather, they are presented as historical witnesses, underscoring their high value as elements of olfactory heritage.

**Re-edition** (sometimes referred to as “reformulation” by industry professionals) may appear similar to *re-weighing* in that it begins with an original formula. However, it differs fundamentally in its purpose, as its goal is to adapt the fragrance to the evolving standards and requirements of the contemporary perfume industry. This involves reworking the original formula to ensure compliance with current regulations, typically for commercial release. Many raw materials once used in perfumery are now restricted or banned, often due to concerns about skin allergies or other health risks, which means that the palette available to perfumers today is markedly different from a century ago. While IFRA (International Fragrance Association) ^9^ guidelines regarding toxicity generally do not apply to historical reconstitutions that are neither made to be worn nor sold, they are strictly enforced for all products intended for the market.

This key distinction justifies the use of a separate term in the NOMEN classification and highlights the significant influence that a commercial objective can have on the reconstitution process. ^10^ The constraints imposed by marketing (such as sanitary, regulatory, industrial, and promotional considerations) mean that olfactory fidelity to the original fragrance is no longer the principal concern.

***Adapting***

***Adaptation*** refers to a specific perfume for which the perfumer possesses a *recipe* (or several variations thereof) that contains imprecise information regarding raw materials, proportions, and techniques. To the existing indications, the perfumer must use their knowledge and experience to add some interpretation, experimentation, and creative input. These recipes often require the collaboration of historians, particularly to trace the techniques and contextual practices used at the time the recipe was written.

The term *adaptation* applies primarily to pre-1880 perfumery, as well as to domestic or artisanal perfumery practices (such as homemade preparations, scented sachets, etc.). The distinction between *re-weighing* and *adaptation* hinges on the difference between a *formula* and a *recipe*. A formula is understood as a precise, rationalised list of raw materials, complete with exact measurements (typically in grams), while a recipe is more akin to a set of “instructions for use” which generally lack a standardised structure or technical precision.

Adaptations are common, but they require a rigorous and methodical approach. The process is often complex, and the outcomes can vary significantly in their fidelity to the original. The older the recipe, the greater the degree of uncertainty — from identifying specific ingredients, to understanding historical extraction methods and sourcing natural materials as they were obtained in the past. In 1996, the Osmothèque — Conservatoire International des Parfums undertook the reconstitution of the "Royal Perfume," based on a recipe cited by Pliny the Elder in his *Natural History* (Book XIII, Chapter 2). The recipe lists 27 ingredients but provides no quantities for them, making the task particularly complex. Jean Kerléo, perfumer and the founder of the Osmothèque, spent over a year working on this adaptation. His first task was to identify the ingredients, then he had to source them, and finally conduct numerous trials, experimenting with different proportions of raw materials. This adaptation prompted extensive research and raised important questions about Roman perfumery, the cultural reception of scents in antiquity, and the potential differences in aesthetic perceptions between ancient Roman perfumers and contemporary ones. ^11^

 The *Seplasia* project, named after the marketplace in Capua where perfumers once made and sold their creations, was launched in 2011 by Jean-Pierre Brun and Xavier Fernandez. It led to the reconstitution of a jasmine perfume based on the recipe for *Iasmelaion*, as described by Dioscorides in *De Materia Medica* (Book I, §77). Following the recipe, jasmine flowers were macerated in oil, after which additional ingredients (myrrh, saffron, and cinnamon) were incorporated. The ancient method was reproduced as faithfully as possible. It is worth noting that this project also drew on chemical analyses of residues found during archaeological excavations, thus placing it within the *reconstruction* category of the NOMEN classification, while also relying on literary and iconographic sources, which align with the *transposition* category. ^12^ This example illustrates the fluid boundaries and complementarity between the different methodological approaches.

A more recent example of *adaptation* is the previously-mentioned *Durare* project, led by Marjolijn Bol at Utrecht University. As part of this initiative, historian Ineke Huysman (Huygens ING/NL Lab) undertook the reconstitution of a perfume based on the letters of Constantijn Huygens (1596–1687), the Dutch diplomat, painter, poet, scientist, composer (and father of Christiaan Huygens) who was also a passionate amateur perfumer. From among the more than 150 recipes mentioned in his correspondence, the team selected one titled *“My mother’s perfumed water”,* which seemed particularly meaningful due to its personal and emotional dimension, reflecting Huygens’ close relationship with his mother. The team attempted to adapt the recipe as faithfully as possible at Utrecht University’s ArtLab. However, the numerous ambiguities in the original instructions ultimately led them to pose new questions about the ingredients, techniques, and broader context of 17th-century perfumery. ^13^ This example underscores how, by placing researchers in direct contact with the material and sensory dimensions of their subject, reconstitution projects ^14^ often open up new avenues of inquiry and generate fresh historical questions. ^15^

These *adaptation* projects are currently multiplying all over the world. ^16^ Since 2021, Sean Coughlin, a researcher at the Czech Academy of Sciences in Prague, has been leading a five-year initiative entitled “Alchemies of Scent” ^17^ The project’s main goal is to reconstitute five perfumes from the Greco-Egyptian period in order to better understand the cultural and technical exchanges which occurred between Greece and Egypt during the Ptolemaic era. Two additional objectives are to develop a lexicon of Greco-Egyptian perfumery and to expand scholarly knowledge of ancient perfumery practices.

Reconstitution is approached here as a form of experimental archaeology — a means of accessing the knowledge and gestures embedded in ancient craftsmanship. The project team refers to their method as *replication,* ^18^ highlighting the degree of interpretation always required when reading ancient recipes and the many variations that result from it. Fragrance outcomes depend not only on the listed ingredients, but also on their quality, the context of production (i.e., the time and place), and the skill of the perfumer. Therefore, several versions of each recipe are tested. Each year of the project is devoted to achieving a new creation: the production of *stakté* (an oil extracted from myrrh), recreating the Mendésian perfume, making *metopion*, the production of *sousinon*, and finally, creating an essential oil by distillation. This work is being carried out by a multidisciplinary research team composed of historians of science, chemists, and Egyptologists. At the time of writing, however, it appears that no professional perfumers have been involved. The same international group is also working on an *adaptation* of a Mesopotamian perfume attributed to Tapputti, a female perfumer at the Assyrian court in the 13th century BCE, whose partial recipe has survived on a cuneiform tablet.

When one or more of the raw materials listed in a historical recipe cannot be located or even precisely identified, the perfumer’s approach typically involves approximating what they believe to be the materials and techniques of the time. This process inevitably includes a degree of trial and error, as well as personal interpretation, often resulting in multiple variants of the same perfume. These variants may also reflect a conscious effort to align the final scent with the olfactory preferences of the present day.

***Updating*** (“Actualisation” in French) refers to the process of following a historical recipe while taking into account modern reception and evolving public tastes. The goal is to bring an old perfume *up to date* by offering a version that resonates with contemporary visitors, rather than presenting them with a strict, historically faithful reconstruction. This updating process may result from the perfumer’s own training and aesthetic biases, but it can also serve an educational purpose.

Changes in scent perception over time, which are shaped by shifting trends in perfumery and a general lowering of olfactory tolerance thresholds ( Corbin, 1982), can be somatically conveyed to today’s audiences. Visitors may find certain smells unpleasant or overwhelming, even though they would not have been perceived that way by people in the past. In this sense, “updating” can act as a kind of *olfactory translation*, revealing nuances and subtleties that might otherwise be lost, or making historical scents more accessible to the modern nose. Conversely, updates can also be used to highlight a feature of the original perfume that was striking at the time of its creation but has since become commonplace, by intentionally amplifying that characteristic to bring it to the attention of contemporary audiences. When updates are intended for commercial use, it is important to emphasise that they are always approximations. In addition to adapting to current tastes, these versions must also conform to today’s regulatory standards: as has been mentioned above, many ingredients originally used in historical formulas are now banned or restricted. As a result, perfumers are often obliged to modify the composition, replacing certain materials with modern substitutes, while striving to remain as faithful as possible to the spirit of the original fragrance.

***Reconstructing***

***Reconstruction*** refers to the process of recreating a fragrance based on the analysis of physical residues, using scientific techniques such as chemistry, archaeobotany, and chromatography. This approach relies heavily on chemical analysis, gas chromatography coupled with mass spectrometry (GC-MS) in particular, to produce the most accurate possible inventory of the molecules present in a given sample. Archaeobotany can also provide valuable insights into the presence of specific plants, as well as the chemical markers of their degradation over time. Reconstruction may sometimes be combined with *adaptation*, particularly when a historical recipe is available alongside the physical sample.

One of the earliest examples of this approach involves the so-called *“Cleopatra’s perfume.”* In 2012, archaeologists Robert Littman and Jay Silverstein, both professors at the University of Hawaii, discovered an ancient perfume workshop dating back to the 3rd century BCE at the site of the ancient Egyptian city of Tell El-Timai. Among the finds were several glass bottles, some of which contained dried residues. The analysis of these materials led to the identification of several ingredients.

A PhD student in Egyptology, Dora Goldsmith (Berlin), and Sean Coughlin (Prague), were commissioned to recreate the perfume, using both the analytical data and recipes found in ancient Greek texts. The resulting fragrance contained myrrh, cardamom, cinnamon, and olive oil, and was showcased in an exhibition at the National Geographic Museum in Washington, D.C. In these examples, many questions remain. ^19^ In a similar vein, in another study published on August 31, 2023 in the journal *Scientific Reports*, archaeo-chemist Barbara Huber, in collaboration with a team of geo-anthropologists from the Max Planck Institute (MPI), analysed residues found on two canopic jars dating to around 1450 BCE. Using a combination of advanced chemical analysis techniques, she identified the substances used in the embalming of Senetnay, the nursemaid of the future Pharaoh Amenhotep II and attendant to Thutmose III. The jars, held at the August Kestner Museum in Hanover (Germany), revealed a complex blend used by priests to prepare someone for the journey to the afterlife: beeswax, vegetable oils, animal fats, bitumen, resin from pine family plants (likely larch), dammar or pistachio resin, and an unidentified balsamic substance.

Although no formal *reconstruction* has yet been attempted, an *evocation* (see below) titled "Scent of Eternity", designed by scent-designer Carole Calvez (Iris & Morphée), was created based on these findings. This fragrance draws upon both the analytical data and secondary sources related to mummification practices of the period, as well as the accumulated knowledge of researchers regarding the olfactory complexity of environments surrounding ancient mummies ( Huber *et al.*, 2023).

Further examples of *reconstruction* can also be found in more recent historical contexts. In a project conducted with scent historian Annick Le Guérer, perfumer Dominique Ropion used GC-MS analysis to study the contents of a perfume bottle discovered in the bathroom of writer George Sand (1804-1876). Based on the findings, Ropion recreated the fragrance, which was deposited at the Osmothèque in 2011. ^20^ Looking ahead, the development of Artificial Intelligence, and particularly its potential to identify degraded molecules, may further enhance the power, precision, and possibilities of olfactory reconstruction.

***Transposing***

***Transposition*** refers to the creation of an olfactory composition intended to evoke elements (either implicitly or explicitly olfactory) found in a literary or pictorial representation. The goal is to bring to life a *represented* scent (such as that of roses depicted in a painting) or an *imagined* olfactory environment, like the smell of the Battle of Waterloo or an Amsterdam canal. The objective is not to reproduce a historically attested scent, but to suggest what the scene might have smelled like, whether it has been derived from a text or an image. While heavily reliant on the creative interpretation of the perfumer, *transposition* also draws upon historical sources and must meet a mimetic aim: to simulate a plausible olfactory reality.

From April to July 2022, the Prado Museum in Madrid hosted an exhibition entitled *The Essence of a Painting. An Olfactory Exhibition*, centred around *The Sense of Smell* (1617–18) by Jan Brueghel the Elder and Peter Paul Rubens. The painting, which features over 80 plants and flowers, as well as animals with a strong sense of smell such as dogs and guinea pigs, inspired the curators to include scent as a sensory complement to the visual artwork. The depiction of perfumed gloves and fragrance-related objects further encouraged the idea of recreating a historical glove scent. Perfumer Gregorio Sola (Puig) therefore developed ten scents for the exhibition, which were diffused into the gallery through four scent dispensers. He also created a free *adaptation* of a 1696 recipe for scented gloves. A commercial version of this scent has since been released, making it an example of *updating* (see above). In another instance of *transposition*, the Odeuropa research group created an olfactory composition inspired by Jan van der Heyden’s painting *View of Oudezijds Voorburgwal with the Oude Kerk* (old church) *in Amsterdam* (1670), housed at the Mauritshuis Museum in The Hague. Here, the perfumer used visual and contextual clues to reconstruct what 17th-century passers-by might have smelled along the canal. The resulting fragrance blended impressions of damp stone, animal waste, and blooming linden trees.

Due to its inherent flexibility, ***transposition*** can result in a wide range of interpretations, some leaning closer to *adaptation*, and others to *evocation*, depending on the nature and specificity of the sources used. ^21^ To account for this diversity, the NOMEN classification includes a subcategory: *atmospherization*.

*Atmospherization* refers to a specific form of transposition focused on the smell of places. It aims to recreate the olfactory atmosphere of a particular space at a given moment in history, using a variety of sources, which can include textual descriptions, visual depictions, and historical data, sometimes also supplemented by modern techniques such as headspace analysis. Depending on the availability and type of source material, *atmospherization* may align more closely with either *reconstruction* or *transposition*.

Several *atmospherization* projects have emerged from the European Odeuropa initiative, which places olfactory heritage at the heart of its research. One notable example is the “Beuning Room”, which is described as *“an olfactory transcription of an 18th-century Amsterdam bourgeois interior”.* This composition includes three distinct olfactory facets representing the room itself, the home, and the street outside — a reflection on the sensory overlap between private and public spaces in the urban environment of the time.

In 2024, a workshop at the École Polytechnique Fédérale de Lausanne (EPFL) digitised a 19th-century panorama depicting the Battle of Morat — a pivotal episode in Swiss history in which the Confederate armies faced Charles the Bold in 1476. The public presentation of this immersive digital experience included visual, auditory, and olfactory elements. Visitors were equipped with *“scented necklaces”* that released the smells of blood, sweat, and horse and goat dung, immersing them in the olfactory atmosphere of the battlefield. These scents were synchronised with the visual cues of the canvas, establishing an immediate mimetic relationship between what could be seen and what could be smelled, thereby intensifying the viewer’s physical and emotional engagement with the panorama. Interestingly, the initial inspiration for this *atmospherization* reportedly came from 19th-century visitor complaints, as some viewers of that time expressed disappointment that the original panorama lacked scents which, they believed, hindered full immersion. While the integration of scent into exhibition contexts remains a relatively recent step, it thus responds to a much older desire: the yearning for a multisensory encounter with the past. ^22^

***Evoking***

***Evocation*** refers to a work of imagination by a contemporary perfumer-creator, inspired by a work from the past. It relies almost entirely on the perfumer’s own artistic vision, with historical references serving merely as points of departure or conceptual stimuli. The category encompasses original compositions that express subjective and personal interpretations of a past element. Unlike *transposition*, *evocation* does not aim for mimetic fidelity; instead, it openly embraces the mediation of the perfumer’s imagination and emotional response to history.

While past influences can subtly inform any olfactory creation, what distinguishes *evocation* is the explicit acknowledgment of historical inspiration and its central role in the creative process. It is a category that encompasses a broad spectrum of compositions and fragrances, reflecting varied and often deeply subjective interpretations of the past. ^23^ The sources from which the perfumer works can be varied in nature.

An example of this approach can be found in a project from the Odeuropa research group in 2024. ^24^ A perfumer drew inspiration from Martin Schaffner’s painting *Anastasis/Christ in Limbo* (1549, Ulm Museum, Germany) to create a scent evoking the imagined atmosphere of Hell as shaped by the collective European imagination. The resulting fragrance conjures up burning flesh, decomposition, dampness, flickering flame, and faint traces of sulphur — a combination designed to unsettle the recipient, to chill the spine. The goal was to tap into a deep reservoir of symbolic and cultural memory; rather than to recreate something literal, to provoke an instinctive, visceral response. This work differs significantly from the transposition based on *The Sense of Smell* (see above), in which identifiable olfactory elements (flowers, gloves, etc.) are visually present in the painting. In the case of *Anastasis*, the scent does not derive from anything directly pictured; instead, the perfumer starts from the emotional and symbolic resonance of the artwork in order to craft an entirely new, evocative experience.

Another example of *evocation* is when perfumer Carole Calvez set out to recreate the smell of Félicien Rops’ studio for the exhibition *At Work: Artists' Studios in 19th-Century Belgium*, held in Namur between January and March 2024. Collaborating with specialists in the olfactory environments of 19th-century ateliers, she imagined the possible scents present in an artist’s workspace of the time. Her approach combined historical knowledge of odoriferous materials typically found in studios (e.g., glues, pigments, and turpentine) with a more imaginative interpretation of what the atmosphere of a painter’s studio might have been like ( Calvez & Wicky, 2023). A particular sub-form of *evocation* is *illustration*, which differs in that it attempts to *olfactorily transcribe* a specific source, even when the source contains no explicit reference to scent. Examples such as translating the mood of an abstract painting or the emotion of an operatic aria into scent would fall under this category.

This leads to the question of whether such evocations retain a mimetic function. One example that raises this issue can be drawn from the Odeuropa project: Ellsworth Kelly’s painting *Orange Blue* (1964, Ulm Museum, Germany) served as the basis for an olfactory composition. Although the artwork is actually modern, it was treated as a source from the past. The perfumer attempted to render the impression of the painting’s two dominant colours into scent: orange was represented by orange essence and C12 aldehyde, and blue by dihydromyrcenol and calone (as per publicly available notes from IFF). The resulting fragrance is an *illustration* of colour through olfactory means — an associative and interpretive exercise rather than a historical reconstruction. In such borderline cases, the claim to a historical dimension is more tenuous, underscoring the complexity of defining the boundaries of *evocation* within a heritage-oriented framework.

### 4. Graphic representation

**Figure 1.**
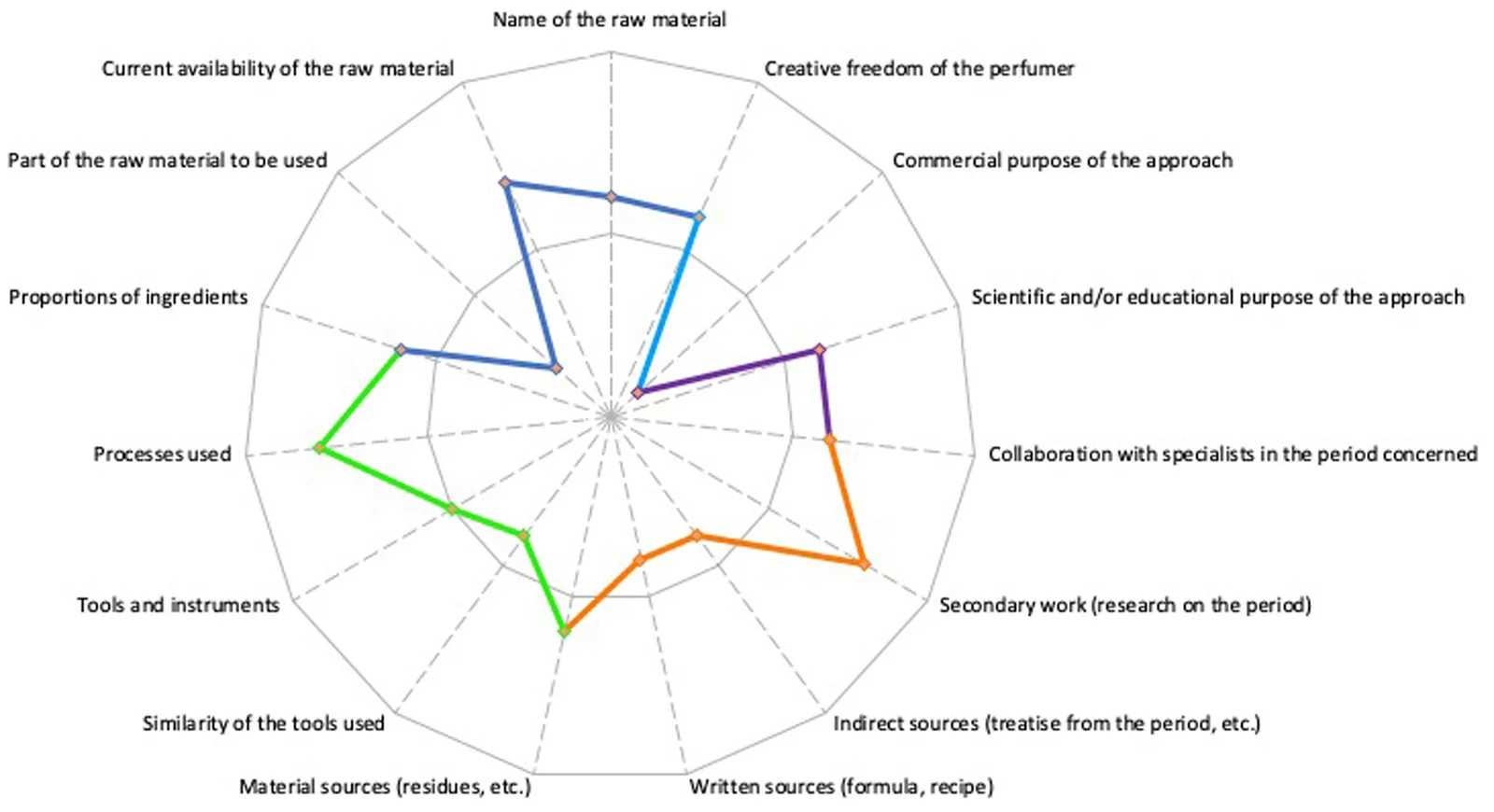
An example of graphical representation.

As we have seen, the NOMEN classification comprises five distinct categories: *Re-weighing*, *Adaptation*, *Reconstruction*, *Transposition*, and *Evocation*. It is structured according to a dual characterisation model: both textual and schematic. Alongside the lexical descriptions, we propose a visual representation system that enables users to quickly and clearly grasp the “profile” of each olfactory composition, thus facilitating comparison both across and within categories. To achieve this, we have developed a Kiviat diagram (or radar chart) model based on fifteen criteria grouped into thematic quadrants (see the Figure 1– Figure 5 below). The upper-left quadrant focuses on *raw materials*, while the lower quadrant contains *reconstitution techniques*. The two right-hand quadrants pertain to the *intentions* behind the project and the *types of external sources* involved. It is important to note that the boundaries between these segments are not rigid; for example, the faithful recreation of a historical recipe may require a degree of creative interpretation, while an artistic evocation that claims historical value often involves thoughtful consideration of materials or techniques. The purpose of the diagram is, therefore, not to rigidly categorise, but to highlight dominant trends and to sketch a characteristic “profile” of each olfactory work, based on the available information.

**Figure 2.**
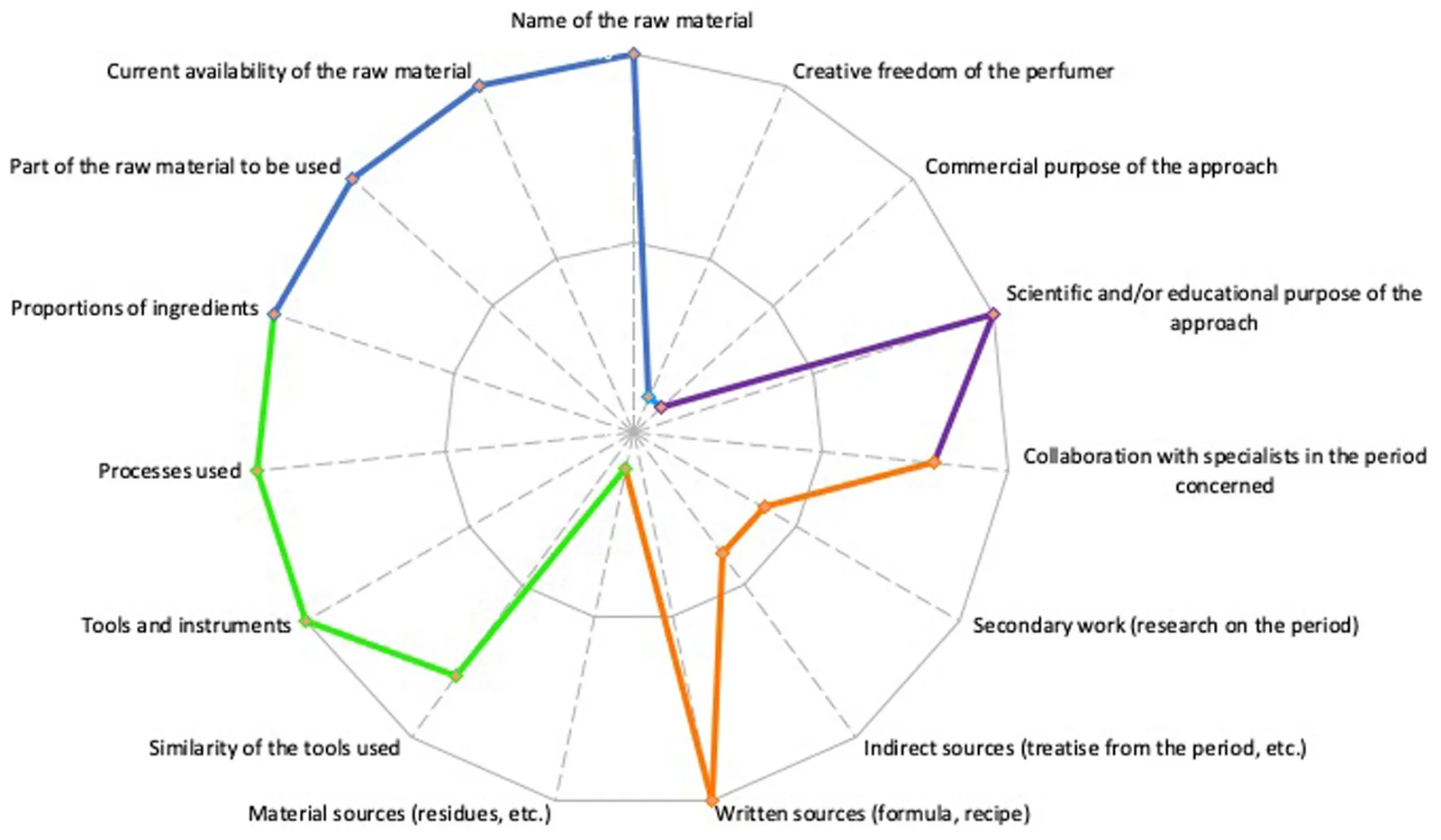
An example of *re-weighing* (L'Origan).

**Figure 3.**
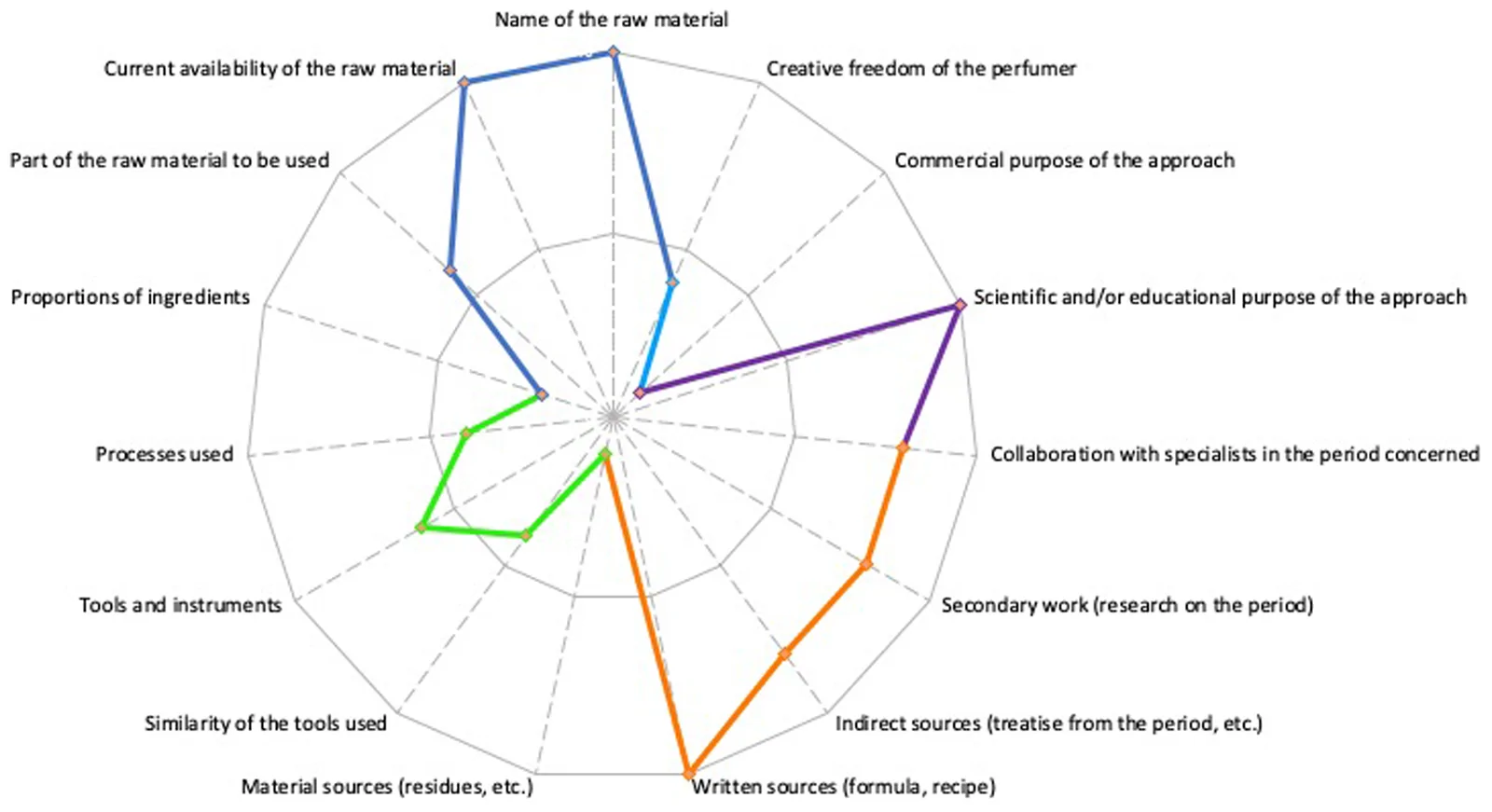
An example of *adaptation* (Royal Perfume).

**Figure 4.**
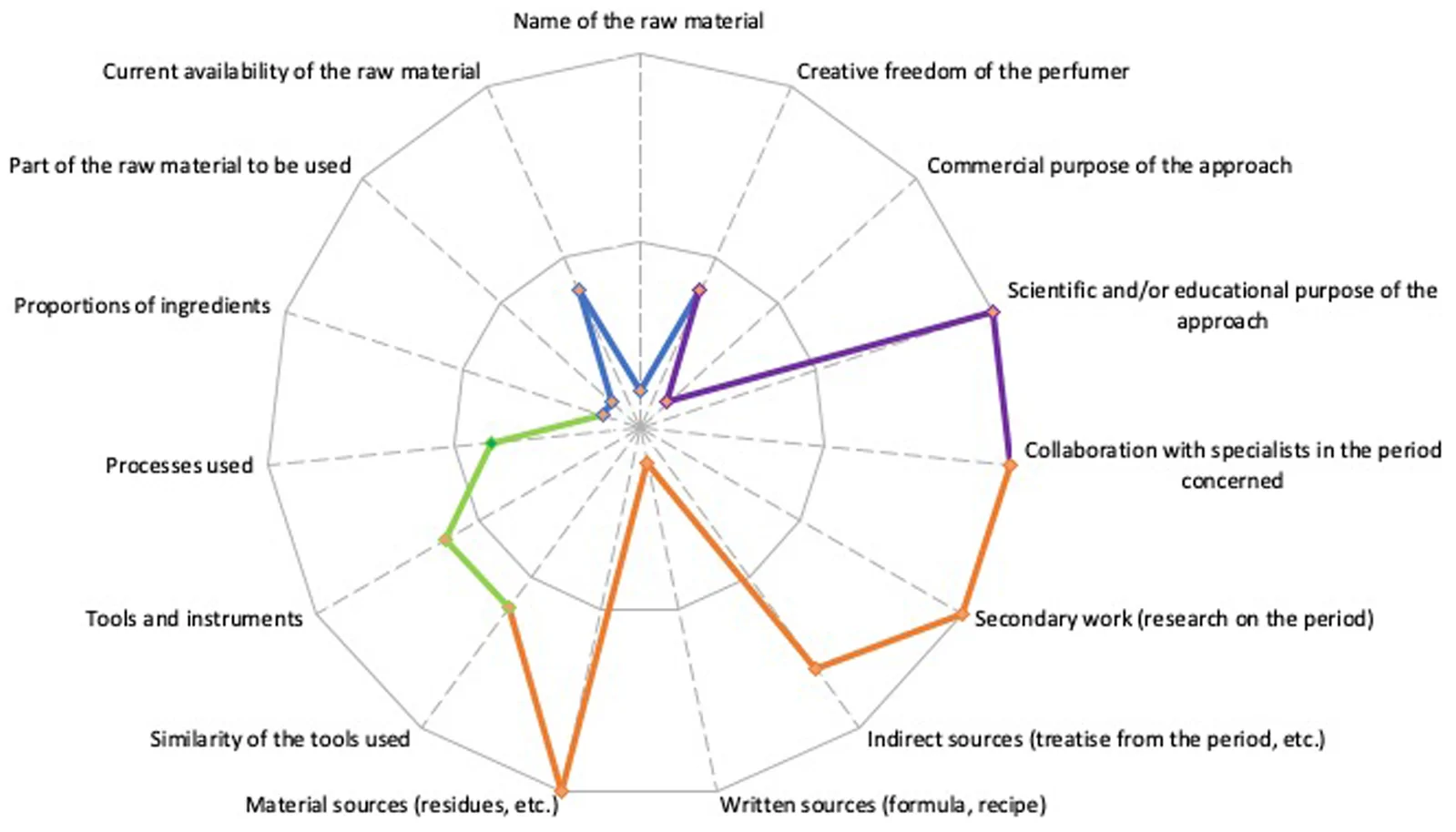
An example of *reconstruction*.

**Figure 5.**
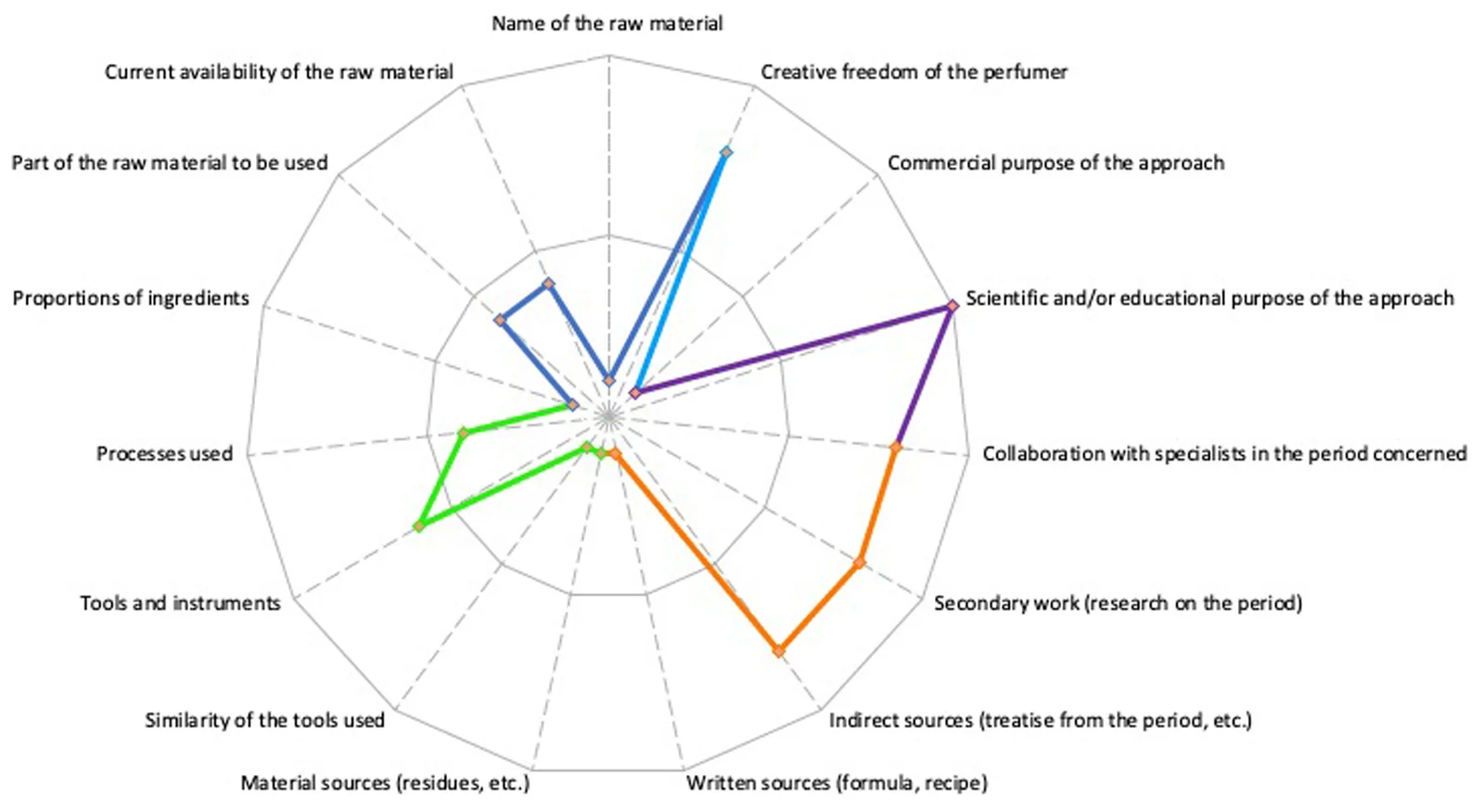
An example of *evocation* (Hell).

The criteria used for the segments are as follows:

**Table:** 

Top left quarter	Top right quarter
Name of raw material (materials and extraction method, specific or not) • Exact name 10/10 • Name of raw material without further details 8/10 • Vague reference to raw material 6/10 • Not relevant 1/10	Scientific and/or educational purpose of the approach • Scientific and educational purpose 10/10 • Scientific and educational purpose mixed with a commercial purpose 6/10 • Not relevant 1/10
Current existence of the raw material. • The raw material still exists and can be used 10/10 • The material no longer exists in its current form and must be replaced 4/10 • Not applicable 1/10	Commercial purpose of the approach • Commercial purpose 10/10 • Non-commercial purpose 1/10
Specification of the part of the raw material to be used (e.g., leaf, flower, or root of the same plant) • The part of the raw material to be used is precisely specified 10/10 • Due to a lack of precision, interpretation is based on a comparative element 8/10 • Due to a lack of precision, interpretation is an absolute 4/10 • Not applicable 1/10	Creative freedom of the perfumer • Complete freedom 10/10 • Partial freedom 6/10 • No creative freedom 1/10
Proportion of ingredients • The quantity is indicated precisely 10/10 • The quantity is indicated vaguely 8/10 • The quantity is not specified, so the interpretation is absolute 4/10 • Not relevant 1/10	
Lower left quarter	Lower right quarter
Processes used • Techniques are specified in full 10/10 • Some techniques are not specified and require interpretation 8/10 • No techniques are specified, so interpretation is absolute 6/10 • Not relevant 1/10	Material sources (residue, etc.) • Residue that can be used 10/10 • Traces provide partial information 6/10 • No residue 1/10
Tools and instruments • All tools are specified 10/10 • Some tools are not specified and require interpretation 8/10 • No tools are indicated, so interpretation is absolute 4/10 • Not relevant 1/10	Written sources (formula, recipe) • Complete formula or recipe 10/10 • Partial formula or recipe 6/10 • No formula or recipe 1/10
Similarity of tools used • All tools are identical 10/10 • Some tools are not identical and have been replaced 8/10 • No tools are identical and all have been replaced 1/10	Indirect sources (treatise from the period, etc.) • Research in contemporary perfume sources to fill in all gaps 10/10 • Research in contemporary perfume sources to fill in some gaps 6/10 • No specific research 1/10
	Secondary work (research on the period) • Research in existing works to fill all gaps 10/10 • Research in existing works to fill some gaps 6/10 • No specific research 1/10
	Collaboration with specialists in the period concerned • Collaboration with specialists in various disciplines 10/10 • Partial collaboration 4/10 • No specific collaboration 1/10

Each criterion is assigned a score from 1 to 10, allowing its relative importance within the context of the specific project to be represented graphically.

**Example of Graphical Representation:**

Comparing the graphs provides a quick visual overview of the differences between several types of reconstitutions.

Examples: of *re-weighing* (L'Origan), of *adaptation* (Royal Perfume), of *reconstruction,* of *evocation* (Hell)

 It should be emphasised that these graphical representations are approximations, and may occasionally lead to misinterpretation due to simplifications or distortions introduced by the arbitrary lines connecting the data points. Despite this limitation, they remain valuable tools, as they facilitate comparative analysis between different olfactory creations and help to visualise the defining characteristics of each category identified in the NOMEN classification. In particular, these graphs prove effective in the context of public engagement and mediation, especially when conveying the diversity and complexity of scent compositions created with a historical purpose or dimension.

To conclude, we have chosen to take a step back and reflect on olfactory compositions with a historical dimension from a philosophical perspective. Chantal Jaquet (2010), a member of the Osmothèque’s Scientific Committee and Professor Emeritus of Philosophy at the University of Paris 1 Panthéon-Sorbonne, graciously agreed to share her thoughts on the subject with us.

## Conclusion - In search of lost scents: the reasons behind a new passion

The NOMEN project reflects the spirit of our time, its concerns and focal interests.

The need to draw distinctions and establish a nomenclature in the field of historical fragrance reconstruction arises precisely from the proliferation of initiatives currently underway and the wide variety of methods and techniques being employed within them, a situation that sometimes leaves us wondering whether we are not being *led by the nose*.

Whether they are museum-based or commercial, scientific or educational, and recreational or instructive, the many ventures devoted to recreating the scents and perfumes of the past clearly reflect a present-day enthusiasm. This enthusiasm is shared both by perfumers, who seek inspiration to create new masterpieces, and by the general public, who flock to Versailles to follow *In the Wake of the Queen* and inhale the perfume known as *Marie Antoinette’s scent*, or who rush to sample the *Queen of Hungary’s water* along the *Promenade des Parfums* in Chamerolles.

Without quite becoming a fully-fledged social phenomenon, this fresh olfactory curiosity is undoubtedly in tune with the spirit of the times, and invites us to ask: what exactly sets our noses running? Regardless of the scientific rigour or intrinsic quality of these reconstitutions, we are entitled to ask: why do they fascinate us so deeply? What do they reveal about us and about our era?

### The counter-proustian olfactory turn

If we were to characterise the fragrant spirit of our time through the lens of these experiments, we would likely highlight what could be called a *counter-Proustian olfactory turn*. The 20th century was unmistakably Proustian, celebrating as it did the evocative power of scent as a trigger of memory, with the madeleine’s aroma becoming the overused archetype, *ad nauseam*. In contrast, the 21st century appears to be reenacting that olfactory scene in reverse. We are no longer seeking to retrieve lost time *through* a scent, but rather to recover *lost scents* through time. In other words, scent is no longer the medium of memory; memory has become the medium of scent. Indeed, perfume reconstruction is now grounded in the tangible traces of the past that remain accessible to us: complete historical formulas detailing exact ingredient proportions, fragmentary recipes, indirect literary and historical references, and the molecules preserved in fabrics, objects, walls, or architectural surfaces. Thus, the classical question of *the scent of memory* is increasingly giving way to a new question: the memory of scent.

This *counter-Proustian* turn is not about opposing the author of *In Search of Lost Time* but about leaning on him to dig deeper into the domains of memory (and overturn them), by no longer starting from fragrant reminiscences, but from total or partial olfactory amnesia. It is therefore less about consciously countering him than about being *against him, very close against him*, to parody Sacha Guitry’s famous phrase. We are thus dealing with a *“contre, tout contre Proust”* ^25^ approach which uses him as a point of departure to head in the opposite direction. To understand the rationale behind this olfactory shift, one that makes perfume the central object of memory research rather than a mere accessory, we must first pose transcendental questions about the conditions that make such a project possible.

### Material and technical conditions

More generally, to fully pursue a passion, one must first have the material means to do so. This is why it is essential not to separate the *why* from the *how*, and to consider the technical systems and tools that are the *sine qua non* for the emergence of an interest in fragrance reconstructions. Given the current proliferation of such initiatives, it would be risky to quickly assume that this enthusiasm is a unique trait of our time — and, by extension, that previous generations simply did not care. Certainly, the historical marginalisation of the sense of smell in many cultures and civilizations has often favoured the sanitisation and eradication of odours over their preservation or reconstitution. Yet this has never extinguished the love of perfume — whether sacred or profane. The fact that fragrance formulas were often jealously preserved, precisely so they could be recreated over time, bears witness to this enduring fascination.

The challenge faced by our predecessors, however, was their lack of effective ways to capture and recreate scent due to its ephemeral nature. It is likely that our ancestors, just as curious as we are about the mysterious fragrance of Alexander the Great (which, legend has it, smelled divine) or Cleopatra’s seductive perfumes, would have rushed to smell them too *if* they had possessed the means to reconstruct them. The advent of modern chemistry and molecular analysis, including innovations like headspace technology and gas chromatography coupled with mass spectrometry (GC-MS), has fundamentally changed the equation and opened up vast new possibilities. These techniques, which allow us to separate molecules and identify the volatile components of a fragrance (or its trace residues on clothing, objects, or architectural surfaces), have made it possible to attempt reconstructions that go beyond mere imaginative speculation in the absence of complete historical formulas.

It should be acknowledged that these powerful tools have some limitations, especially in terms of coping with the volatility of certain molecules, which evaporate more quickly than others. As a result, reconstructions often capture more of the base notes than the top notes of ancient perfumes, and can offer only a partial impression rather than a full recreation. The degradation of odorant molecules over time must also be considered. These techniques cannot compensate for the disappearance of certain raw materials, or for the lack of information about exact ingredient proportions as noted earlier in the discussion of the NOMEN categories. Nevertheless, we must acknowledge that the recent rise in interest around olfactory reconstructions is closely tied to the development of olfactometry and other cutting-edge technologies that now offer a glimpse of what once seemed irrevocably lost to time. In that sense, this trend is less the expression of a novelty than the revival of an ancient desire, made newly tangible by appropriate technical means.

### The theoretical reasons behind the enthusiasm for olfactory reconstitutions

 However, having the means to satisfy a desire is not enough by itself to explain the emergence of a strong and sustained interest; there must also be compelling motivations to cultivate it. Without claiming to provide an exhaustive list, we can identify two main types of reasons behind this growing trend toward the reconstruction of historical perfumes: one theoretical and cognitive, and the other aesthetic and artistic.

The first reason is linked to the growing interest in osmology (the scientific and historical study of scent) both in its contemporary forms and in its traces from the past. This theoretical interest is closely tied to the rise of sensory anthropology, and more broadly to the ongoing rehabilitation of the senses — particularly smell, which, as is well known, had long been neglected and ranked lowest in the traditional sensory hierarchy. If nothing fragrant is now foreign to us, it is only natural that we will wish to seek out lost scents and enrich the historical and scientific understanding of perfume through reconstruction. In short, this renewed enthusiasm is one of the clear symptoms of the new epistemological status of the sense of smell in the 21st century. Once a marginal sense, smell has now moved to the forefront of scholarly attention — a shift accelerated by the COVID-19 pandemic, which made the effects of anosmia (loss of smell) starkly obvious and emotionally resonant for many people.

This overall cognitive approach is itself driven by different motivations, depending on the specific disciplines involved, such as the history of perfumery, archaeology, or sensory anthropology. The reasons for undertaking scent reconstructions inevitably vary according to the goals of each field. In perfumery, for instance, the revival of theoretical interest in historical recreations primarily follows a museological and heritage-driven logic, exemplified by the founding of the Osmothèque in Versailles in 1990, which from the outset has focused on faithfully reconstructing perfumes of the past. More recently, the Odeuropa project (2020–2023) sought to build a true *encyclopedia of scent*, cataloguing the smells and perfumes encountered in Europe from the 16th through to the 20th century. This cognitive approach is akin to the work of archivists and museum curators in other domains of knowledge and is not unique in itself. Rather, it represents a belated olfactory normalisation of the broader historical movement toward preserving traces, collecting data, and reconstructing information across multiple media. It is fully aligned with the contemporary logic of the accumulation, digitisation, and dissemination of knowledge — a logic nowadays shaped by artificial memory systems, file storage technologies, and the expansion of digital capitalism.

While this search for olfactory archives also inspires archaeologists, their motivations and objectives differ from the aforementioned ones in important ways. In the archaeological context, reconstruction is driven less by the desire to retrace the history of perfume across the centuries than by the aim of understanding the history of a civilization — its ways of life, its material culture, and the social functions of the objects that surrounded it. This is exemplified by the research conducted by Barbara Huber and other German scholars, who have sought to reconstruct the scents associated with the embalming of ancient Egyptian mummies, the incense burned in Babylonian temples, and the fragrant atmosphere of domestic life from 4,000 years ago — examples discussed above in the section on “reconstruction”. Recognising the central role of fragrance in pharmacopoeia, sacred rituals, seduction practices, and the establishment of social hierarchies, these archaeologists use perfume reconstruction as a means to make the spirit of an era perceptible, by literally offering it to be smelled.

In sensory anthropology, the focus shifts more toward the comparative analysis of cultural constructions of olfactory perception, and to highlighting how judgments and reactions to smells vary across time and context. The reconstruction of ancient perfumes enables us to explore the evolution of tastes and aversions in the realm of scent, revealing how our olfactory perceptions are shaped by customs, habits, and cultural context rather than by our physiology alone. For instance, Barbara Huber notes that in ancient Egypt, fish was considered to have such a foul odour that its corresponding hieroglyph symbolised *stench*. Likewise, someone smelling the *Queen of Hungary’s Water* today might be struck by its strong rosemary note, which is more likely to evoke a kitchen aroma or the sun-scorched scrubland of the Mediterranean than the refreshing promise of a timeless elixir of youth.

In this case, the cognitive interest centres on the history of the cultural construction of the sense of smell. But whether the focus is on the history of odours, the history of civilizations, or the cultural history of the senses, the reconstruction of perfumes ultimately belongs to a gnoseological approach that seeks, in every instance, to build knowledge. Here, the desire to know is the symptom of a growing recognition of the dignity and importance of the olfactory world — past, present, and future.

### Aesthetic and artistic motivations

This cognitive interest, however, is not the only (nor perhaps even the primary) driver behind the burgeoning research in this field. Theoretical curiosity is often accompanied by a strong aesthetic and artistic sensibility, particularly among perfumers and connoisseur audiences. In this context, what motivates the endeavour is not so much the pursuit of perfect, formulaic reconstructions ( *re-weighings*) than the poetic evocation and sensory commemoration of historical or iconic fragrances. Perfumers may seek to recreate scents from the past to nourish their artistic imagination, to discover new models of inspiration, and to draw on the audacity and originality of their predecessors in order to find the strength and confidence to invent fresh olfactory masterpieces of their own.

Though such a process may carry a hint of nostalgia, it is by no means retrograde. It is not a step backward; rather, it is a form of creative renewal and a reclaiming of the past, without which no future is possible — for nothing is created *ex nihilo*. These reconstructions do not follow a purely mimetic logic; they are not mechanical repetitions, slavish copies, or crude forgeries. Like a musical score, a perfume formula cannot be reproduced mechanically; it calls for interpretation, requiring the sensibility, skill, and inventiveness of the performer. In their quest for the lost perfume, perfumers often must display remarkable ingenuity in finding ways to evoke the scent of a natural ingredient that has disappeared, or replace a now-prohibited substance with an ethically or medically acceptable alternative.

In this sense, reconstructions are less reproductions than rediscoveries. And in *rediscovery* [ *retrouvaille* ^26^], we quite literally find the idea of *finding again*, which implies creativity within fidelity. Through such reconstructions, each bearing the unique imprint of its creator’s style, perfumers may stake a claim to a prestigious lineage or assert a bold freedom of interpretation that places them among the greats. In so doing, they inscribe themselves into history, bathed either in the aura of their illustrious forebears, or in the brilliance of their rupture with past conventions of perfume composition.

Whatever the underlying motivations — which can range from a search for inspiration, an artistic statement, or a marketing strategy, to the commemoration of an exceptional event — these reconstructions always serve to celebrate the past; to rescue it from oblivion by making it *tangible to the nose*, or by interpreting it as an olfactory variation on a historical theme. Whether they are faithful or not, these reconstitutions awaken the public imagination and offer the illusion of a long-buried world rising from the bottle, as if by magic. They defy the irreversibility of time and bring pleasure by offering a dreamy fragment of a vanished era — misty, vaporous, and vaporised.

To *recreate in order to re-enchant* is the ultimate impulse behind these efforts. This hedonistic memorial pursuit may well be one of the most powerful forces driving the search for lost fragrances. In such moments of experiencing them, knowledge is joined by delight, and death is defied by the resurrection of a scent — a fleeting, fragrant fragment of eternity.

Despite the difference in cultural context, it may be illuminating to compare this form of pleasure to the experience described by a Japanese master of *kōdō* from the Shino school, who confided in a private conversation that, through *listening to jinkō* (a term referring to the inhalation of the breath of precious aromatic woods) he feels deeply united with all those who delighted in the same fragrances in times past. He described a sense of belonging to an olfactory community that transcends time, and of sharing the joy of that unique, fleeting moment with them. To feel connected across centuries to a chain of past generations, and to sense their presence through a perfume that envelops us and fuses us with them may be one of the keys to the mysterious appeal of restored fragrances.

Ultimately, the fascination with reconstructed historical perfumes is nourished by a desire for a kind of knowledge that is also a *co-nascence*, a shared birth that erases the boundaries of space and time between generations. It carries both memorial and emotional dimensions, in keeping with the very nature of olfactory sensitivity. Even if cognitive, aesthetic, and emotional motivations do not fully account for the appeal of such reconstructions, one thing remains clear: that olfactory reconstitution is neither lost time, nor recovered time — it is unknown time, given to be felt, without ever having been lived.

In an age where one piece of information displaces the next, where images scroll endlessly across screens, and where everything is marked by rapid evanescence, the nose anchors us in the invisible depth of a restored scent, that reconnects us to the world and to the shared human experience. To *reconstitute oneself through reconstitution* is perhaps the deeper reason behind our pursuit of the embalmed bottles of the past.

## Ethics and consent

Ethical approval and consent were not required.

## Data Availability

Not Applicable. This article provides a model for analysing odorous composition with a historical dimension.
