# Supplementary material for: NOMEN — A classification of methods used to create odourous compositions with a historical dimension: re-weighing, adapting, reconstructing, transposing, evoking
Source: Open Res Eur. 2025 Dec 10;5:383. [Version 1] doi: 10.12688/openreseurope.21121.1 (PMC13601792; doi:10.12688/openreseurope.21121.1)
Supplement: Peer review history

# Reviewer report: Reviewer response for version 1

**DOI:** 10.21956/openreseurope.22846.r66103

**Published:** 2026-01-16

**Authors:** Cecilia Bembibre

**Affiliations:** 1 University College London, London, England, UK

 The article provides a useful classification of approaches for recreating or reinterpreting smells associated with what the authors describe as “vanished” scents or atmospheres, understood as historical olfactory experiences that are no longer directly accessible. This has the potential to be a timely and valuable contribution, particularly given the growing number of museum, research and commercial initiatives working with historic smells.

 From the keywords through to multiple passages in the text, the authors consistently position the NOMEN framework as a contribution to olfactory heritage, referring to a “rich but often overlooked olfactory heritage,” to the Osmothèque as a pioneer in its preservation, and to the classification as a heritage-oriented effort to preserve the memory of meaningful past scents. Currently, however, it is not entirely clear the way olfactory heritage is conceptualised by the authors in relation to history. A brief exploration of this distinction would strengthen the conceptual grounding of the framework, for example by clarifying whether the classification is intended to address the 'recovery' of the past or the contemporary processes through which selected elements of the past are mobilised, curated and valued as heritage.

 This issue becomes more pronounced in relation to the authors’ claim that “the aim of this classification is, therefore, also heritage-oriented in that it seeks to acknowledge and document the diverse approaches used to preserve the memory of meaningful scents from the past.” While this is an important ambition, the paper would benefit from greater contextualisation of what is meant by memory in this setting. It remains unclear whose memory is being preserved and on what basis certain scents are deemed meaningful.

 The framework provides limited reflection on point of view and on the position of the “sniffer,” whether this is the perfumer, the curator or the visitor. This raises broader questions about how embodied experience, cultural framing and power relations shape olfactory representations, and the authors are encouraged to address these dimensions more explicitly, to prevent the classification treating odours as transferable technical objects instead of situated experiences whose meanings emerge through practice, memory and interpretation.

 The article also states that the classification is not limited to perfume in the strict sense and that it applies to historical olfactory compositions in the broadest way. In practice, however, the framework remains heavily centred on perfumery. The categories of re-weighing and re-edition are intrinsically perfume-based, and the examples discussed under adaptation, updating and reconstruction also concern perfumes. Only the final two categories, transposition/atmospherisation and evocation, explicitly engage with non-perfume experiences such as historical smellscapes derived from visual sources or multidisciplinary heritage research. It is in these latter categories that the article offers very valuable reflections on the relationships between smells, materiality, intention and temporal distance involved in bringing back historical olfactory experiences. This suggests the need to revisit or qualify the claim that the framework addresses historical smells in their broadest sense.

 A further point concerns the positioning of NOMEN in relation to existing frameworks to represent smells of the past. More explicit engagement would clarify how the present classification aligns with, diverges from or extends earlier approaches, and would help to situate NOMEN more clearly within an emerging field of methodological reflection.

 Finally, the proposed visual representation system using radar charts is a strong element of the paper and has clear potential for wider adoption, given their established use of these charts in both academic research on olfaction and in the flavour and fragrance industry. At present, however, these charts remain largely oriented towards perfume. The authors are encouraged to consider how the system might accommodate differences in the original form of the smell, whether this is a wearable perfume, an ambient installation or a smell emerging from materials or practices. Additional dimensions might include the intended purpose of the approach, such as interpretation, education or inclusivity and accessibility, the degree to which experts and non-experts are involved, and whether the smell is treated as an autonomous object or as an attribute of a material artefact, a practice or a space. In this respect, the classification of olfactory heritage proposed in the Olfactory Heritage Toolkit (Bembibre, C., Leemans, I., & Elpers, S. (2023). The Olfactory Heritage Toolkit. Zenodo. https://doi.org/10.5281/zenodo.10775277) could provide a useful point of reference.

 Overall, the article has considerable value in mapping a rapidly expanding field and in offering a shared vocabulary for describing diverse practices. Its impact would be further strengthened by exploring the relationships between the different methodological approaches to bringing back historic smells and questions of authorship and authenticity in a heritage context, while acknowledging the adjacent body of work concerned with safeguarding smell as part of living heritage, which, although beyond the scope of the article, is nonetheless relevant to situate the framework more fully.

 A few of minor notes:

- There is no Whiffstory Toolkit – there are however two articles with similar titles, one cited (Marx L, Ehrich SC, Tullett W, *et al.*: Making whiffstory: a contemporary re-creation of an early modern scent for perfumed gloves. *Am Hist Rev.* 2022; **127**(2): 881–893.)  and one not cited (Refer Ref no. 1)

 

- I could not find a reference for the commercial release of the Guantes de Ambar scent from Gregorio Sola – it would be helpful to include it.

If any, are all the source data and materials underlying the results available?

No source data required

Is the work original in terms of material and argument?

Yes

Is the work clearly and cogently presented?

Partly

Is the argument persuasive and supported by evidence?

Partly

Does the research article contribute to the cultural, historical, social understanding of the field?

Yes

Does it sufficiently engage with relevant methodologies and secondary literature on the topic?

Yes

Reviewer Expertise:

My expertise is in cultural heritage, with a particular focus on intangible, sensory and olfactory heritage.

I confirm that I have read this submission and believe that I have an appropriate level of expertise to confirm that it is of an acceptable scientific standard, however I have significant reservations, as outlined above.

## References

[1] : Whiffstory. *The American Historical Review* .2022;127(2) : 10.1093/ahr/rhac159849-879 10.1093/ahr/rhac159

# Response

**Published:** 2026-04-27

**Authors:** Isabelle CHAZOT

**Affiliations:** Laboratoire de Recherche Historique Rhone-Alpes, Lyon, Auvergne-Rhône-Alpes, France

We would like to express our sincere thanks to Dr Cecilia Bembibre for her  constructive review of our manuscript. We are particularly grateful for her attention to the heritage dimension of the NOMEN framework and for her invitation to clarify the relationship between history, memory, and olfactory practice. Her comments have helped us strengthen the conceptual grounding of the article and refine its positioning within the broader field of olfactory heritage studies.  

**1. Conceptualisation of olfactory heritage in relation to history** We aknowledge the need to clarify how olfactory heritage is conceptualised in relation to history. In the revised article, we have clarified that NOMEN does not aim to “recover” the past in any straightforward sense, but rather to describe contemporary practices that engage with historically situated materials, narratives, and sensory imaginaries. We now emphasise that historically inspired scent compositions are themselves products of present-day interpretative frameworks and institutional contexts. In this sense, NOMEN addresses the methodologies through which are made attempts to reactivate the past and  re-signify in the present, rather than presuming direct access to historical experience.

**2. Memory and the question of significance** In response to  the reviewer’s invitation to clarify what is meant by “preserving the memory of meaningful scents” and whose memory is at stake., we have revised the relevant passages to avoid any implication of a universal or self-evident notion of meaningfulness. We now specify that the significance attributed to particular scents is culturally situated and historically constructed, and that NOMEN does not determine which scents deserve preservation. Rather, it documents the methodological approaches through which certain scents are created and framed as historically meaningful by researchers, perfumers, and institutions. This clarification aims to avoid essentialising memory and to acknowledge that heritage-making processes inevitably involve acts of selection and interpretation.

**3. Point of view and the role of the “sniffer”**  Regarding the reviewer’s suggestion to address more explicitly the position of the “sniffer,” whether perfumer, curator, or visitor,  we have sought to preserve the focus of the article on methodology rather than expanding into a full discussion of audience reception, which we acknowledge as important but beyond the present scope of our study.  

**4. Perfume-centred framework **  NOMEN’s framework  applies to historical olfactory compositions in the broadest sense. In the revised manuscript, we clarify that NOMEN emerged from expertise developed within the field of perfumery. However, we explicitly state that the framework also encompasses compositions designed to evoke environments, practices, and atmospheres, even when these were not originally perfumes. We acknowledge that these referents are not ontologically equivalent and that their inclusion within a single classificatory model reflects a shared contemporary mode of producing historically oriented scent objects. In the reviewed article, we emphasised that NOMEN focuses specifically on the methodological processes of creating historically oriented scent compositions, whereas other frameworks—including the Olfactory Heritage Toolkit—address broader issues of documentation, preservation, and participatory engagement. This approach is part of an emerging methodological landscape and aims to enrich the discussion by providing opportunities for debate and progress.  

**5. About the graphical charts** We have made it clearer in the revised manuscript that these graphical representations could include additional criteria and be adapted to different users and audiences. This will be an interesting topic to discuss in the future.

# Reviewer report: Reviewer response for version 1

**DOI:** 10.21956/openreseurope.22846.r66102

**Published:** 2026-01-13

**Authors:** Barbara Huber

**Affiliations:** 1 Department of Archaeology, Max Planck Institute of Geoanthropology, Jena, Germany

This article introduces NOMEN, a descriptive classification framework intended to distinguish different methods used to create odorous compositions with a historical dimension, ranging from re-weighing and adaptation to reconstruction, transposition, and evocation. It also proposes a graphical profiling tool designed to visualise and compare the sources, techniques, and intentions underlying such creations.

 Overall, this is an ambitious and timely contribution to the field of olfactory heritage and historically informed scent-making. The authors bring together a rare combination of expertise in perfumery, heritage practice, and scholarly research, and they address a genuine need for greater clarity and transparency in how “historical” scents are produced and communicated. With some further clarification and refinement, the framework has potential to become a useful reference tool for researchers, perfumers, and cultural institutions alike.

 One point that would benefit from being made more explicit is what exactly NOMEN is intended to classify. At times, the paper moves between ancient perfumes recreated as perfumes and then it mentioned scents created to evoke places, atmospheres, or smellscapes. While both clearly belong to contemporary olfactory heritage practice, they are not entirely equivalent, and readers may wonder whether they are meant to be treated in the same way within the framework. A brief clarification of this scope would make the application of NOMEN clearer and help avoid confusion when readers attempt to apply it to their own projects.

 Within the five categories, there appears to be some ambiguity in how they are delineated, and certain methodological overlaps make them difficult to distinguish consistently in practice. Re-weighing is defined very clearly, but the remaining categories would benefit from sharper differentiation. According to the authors, *adaptation* involves recreating a historical perfume from an imprecise or incomplete recipe, requiring interpretation and creative decision-making to fill gaps in ingredients, proportions, or techniques. However, a similar situation often arises when scents are reproduced on the basis of chemical analysis – which is currently in the category *reconstruction*. Ancient organic residues rarely yield a complete molecular profile, and even when analytical data allow a complete recipe to be inferred, substantial uncertainties typically remain, for example regarding missing compounds, or exact ratios. In this sense, *reconstructions* based on chemical data frequently require interpretive choices that closely resemble the adaptive processes described under *adaptation*. The framework would therefore benefit from clarifying how adaptation differs from reconstruction when both approaches involve significant interpretation due to incomplete evidence.

 The category of *reconstruction* would also benefit from further differentiation based on the temporal distance of the material. For example, working with an 18th-century perfume flask that still retains a faint smell allows for a direct sensory comparison between the object and the reconstructed scent. In contrast, for ancient, much older materials, the original odour may be entirely lost, leaving only non-odour-active molecular traces. In such cases, the reconstructed scent is based on inferred recipes derived from characteristic biomarkers rather than on any surviving olfactory reference. These two situations represent qualitatively different levels of reconstruction, yet they are currently grouped under the same category. Related to this point, the category of reconstruction currently encompasses both cases where an odour is partially preserved and cases where it has been completely lost, which risks conflating *re-smelling* (comparison with a surviving scent) and *re-inferring* (reconstruction from molecular traces alone). Making this distinction explicit would clarify what kind of historical access is being claimed in each case.

 The category of *evocation* also appears blurred in certain instances. For example, the work by Carole Calvez on the *Scent of the Afterlife* is described as *evocation* in the paper, yet it draws directly on chemical and archaeological data. This raises the question of whether evocation should be reserved for projects based primarily on imaginative or symbolic interpretation, and whether cases informed by analytical evidence, even when interpreted creatively, might require clearer differentiation. Overall, the boundaries between adaptation, reconstruction, and evocation would benefit from more explicit diagnostic criteria.

 Finally, I occasionally found myself wondering how NOMEN is intended to be used in practice. Many projects I’m aware of combine several approaches at once, for example by drawing on both historical recipes and chemical analyses. It would therefore be helpful to clarify whether projects are expected to fall into a single category or whether overlaps are not only possible but anticipated. Even a short paragraph explaining how to deal with mixed cases would make the framework easier to apply. A simple decision tree or diagnostic checklist could enhance the usability of NOMEN and reduce inconsistent applications.

 Minor comments:

 To my knowledge, the project by Coughlin and Goldsmith mentioned in the section on reconstruction was not based on chemical analysis, but rather on ancient textual sources. While Robert Littman and Jay Silverstein did identify organic residues at the site of Tell el-Timai, the recreation of the so-called “Cleopatra’s perfume” appears to have been based mainly on recipes and descriptions preserved in ancient texts. In this sense, the example may fit more closely within the category of adaptation as defined by the authors. I am aware that chemical analyses may have been conducted by Silverstein, but to my knowledge these results have not yet been formally published. Clarifying this point would help ensure that the example is placed in the most appropriate category.

 The distinction between re-weighing, re-edition (reformulation), and updating is important but may not be immediately clear to readers outside perfumery. A short glossary or summary table could help clarify these relationships.

If any, are all the source data and materials underlying the results available?

No source data required

Is the work original in terms of material and argument?

Yes

Is the work clearly and cogently presented?

Partly

Is the argument persuasive and supported by evidence?

Partly

Does the research article contribute to the cultural, historical, social understanding of the field?

Yes

Does it sufficiently engage with relevant methodologies and secondary literature on the topic?

Yes

Reviewer Expertise:

I'm an archaeo-chemist working in the field of archaeological science. I specialize in the chemical analysis of ancient organic residues, particularly aromatic and spice plants.

I confirm that I have read this submission and believe that I have an appropriate level of expertise to confirm that it is of an acceptable scientific standard, however I have significant reservations, as outlined above.

# Response

**Published:** 2026-04-27

**Authors:** Isabelle CHAZOT

**Affiliations:** Laboratoire de Recherche Historique Rhone-Alpes, Lyon, Auvergne-Rhône-Alpes, France

We would like to express our sincere gratitude to Dr Huber for her careful, technically informed, and constructive review of our manuscript. We are very pleased that she recognized the potential of this classification. We greatly appreciate the clarity with which she identifies methodological ambiguities and the precision of her observations regarding analytical reconstruction, chemical inference, and classificatory consistency. Her comments have significantly helped us refine the NOMEN framework and clarify its practical applicability. We respond below to her main remarks.

 1 **. Scope of NOMEN: perfumes versus broader historical smell compositions** In the revised manuscript, we now clarify more explicitly that NOMEN is intended to classify contemporary olfactory compositions that explicitly claim a historical dimension, whether these concern the recreation of historically attested perfumes or compositions that evoke past environments, practices, or smellscapes. We acknowledge that these referents are not ontologically equivalent.  Their inclusion within a single framework reflects a shared contemporary practice of mobilising historical claims through scent production. This clarification has been added to the section on scope of application. Their inclusion also reflects the need for each “reconstitution” to undergo a process that creates a “fragrance/scent” using today’s molecules or fragrant material as the odorous compositions rely on modern perfumery techniques

 .2 **. Adaptation and reconstruction:  interpretative overlap** We are grateful for the reviewer’s nuanced discussion of the overlap between adaptation and reconstruction, especially in cases where chemical analysis yields incomplete molecular profiles and therefore requires interpretative decision-making. We agree that reconstruction based on analytical data frequently involves choices comparable to those required in adaptation from incomplete recipes. In response, we have clarified that in NOMEN the distinction does not rest solely on the presence or absence of interpretation, since interpretation is inherent in all categories, but rather on the primary methodological claim guiding the composition. We thus stressed the fact that adaptation refers primarily to projects grounded in textual or recipe-based sources that require interpretative completion while reconstruction refers primarily to projects grounded in material or chemical evidence, even when such evidence is partial and demands inferential supplementation. We have added explicit wording to state that categories are assigned according to the dominant evidentiary framework rather than the exclusive use of a single type of source. This aims to preserve analytical clarity while acknowledging hybridity.

**3. Differentiation within the category of reconstruction** We thank the reviewer for highlighting the need to distinguish between cases in which residual odour remains perceptible and cases in which only non-odorant molecular traces survive. This distinction is indeed methodologically significant. In the revised manuscript, we have introduced a clarification within the reconstruction section distinguishing reconstructions based on residual olfactory continuity (where comparison with a surviving scent trace remains possible), and reconstructions based exclusively on inferred molecular profiles without surviving olfactory reference. We have emphasised that these represent different degrees of historical mediation and different types of epistemic access to the past, even though both fall within the broader category of reconstruction.

**4. Evocation and analytically informed projects** In the revised text, we clarify that this example illustrates the fluid boundaries between categories. In this case, while the composition was informed by scientific findings, it did not claim to reconstruct a historically attested recipe, but rather to translate analytical knowledge into a contemporary interpretative composition. We have rephrased the passage to avoid ambiguity regarding its classificatory status and to emphasise that such cases highlight the interpretative nature of the framework. **5. Hybrid projects and practical use of NOMEN** We have clarified that NOMEN does not require exclusive categorisation. Many projects may legitimately display characteristics of several categories. In such cases, the classification reflects the primary methodological orientation while allowing secondary influences to be represented in the visual profile. The radar chart system is particularly suited to rendering such hybridity visible. We have also added a short clarification explaining how mixed cases should be approached in practice

**6. The example of Coughlin and Goldsmith** Regarding the methodological basis of the so-called “Cleopatra’s perfume”, we acknowledge that the published documentation foregrounds textual and historical sources more prominently than chemical analysis, and that the analytical results have not been formally published. In light of this observation, we have revised the description to reflect more accurately the evidentiary basis of the project and to clarify the grounds on which it is situated within the reconstruction category. These revisions highlight the need to provide not only the public but also cultural institutions and researchers with precise information on how the odorant compositions were produced.

**7. Distinction between re-weighing, re-edition, and updating** These distinctions may not be immediately clear to readers outside the field of perfumery. To improve clarity, we have refined the definitions of these categories and strengthened the explanation of their respective objectives. We have also ensured that the differences between fidelity-driven re-weighing and commercially motivated reformulation (re-edition/updating) are stated more explicitly. The case of “updating”  refers to the process of following a historical recipe while taking into account modern reception and evolving public tastes.”

# Reviewer report: Reviewer response for version 1

**DOI:** 10.21956/openreseurope.22846.r66104

**Published:** 2026-01-08

**Authors:** William Tullett

**Affiliations:** 1 University of York, York, England, UK

This is an interesting and important intervention in the field of smell heritage that draws on an interdisciplinary range of expertise within and without academia. It is written by a group with extensive expertise in smell history, in the archiving and curation of perfume history, and in the history of flavour and fragrance science. The following are a series of provocations to this text (given that the reviews are open) that are partly aimed at potential revisions and partly offered for readers to engage with alongside the article itself because in some cases they constitute differences of opinion or interpretation. A lot of the below is concerned with the question of framing - of what this classification system is really trying to classify.

 Before that though, let me start with a quick clarification. The reference to a 'Whiffstory Toolkit' was slightly odd. The Odeuropa project, of which (for purposes of transparency) I was a member, produced two articles in *The American Historical Review *with ‘Whiffstory’ in the title. The first, which is not cited, is titled “Whiffstory: Using Multidisciplinary Methods to Represent the Olfactory Past'’. Despite not being cited, this is the piece that offers a classification of different ways of engaging with the past through smell. However, the cited piece is ‘Making Whiffstory’ which was specifically linked to the publication of a smell in the journal in the form of a scratch and sniff card. We also produced a separate ‘Olfactory Storytelling Toolkit’ (also not cited) as a guide to using smell in museums and heritage settings. This is all to say that there is no such thing as the ‘Whiffstory Toolkit’ and so this requires editing and disambiguation. As an addendum to this point, I thought that the description at the end of the paragraph in question - found squirrelled away in footnote 4 rather than open in the main text - to be rather ungenerous. We are told that a scholar 'attempted' to do something, but offered 'misinterpretations and confusions', without spelling out what those confusions were (in this case a very early career scholar). The result is that the researcher is criticised without offering substantive evidence or justification of that criticism. The footnote also adds nothing to the argument. I would suggest either citing the piece in question and engaging with it directly in the main body of the text or leaving out the footnote entirely. A similar mode of casting doubt without enlightening the reader further is found in the discussion of the work of Goldsmith and Coughlin where we are told ‘In these examples, many questions remain’ but not what those questions are or why they are important (and the cited material here is an online magazine article, hardly the place for detailed scholarly discussions of method?).

 With that out the way, let’s return to the more substantive content and claims of the article and that provocations I’d like to make in response.

 The first provocation concerns the framing of the article's subject matter. It is unclear whether the article is concerned with the Osmothèque's normal focus - perfume - or with smell in a heritage setting more generally. This lack of clarity surfaces at various points, starting with the abstract. The abstract begins "The past decade has seen a proliferation of olfactory compositions and devices claiming a degree of mimetic connection to a vanished scent or atmosphere...". There are a few problems with this statement. The first is that that smell has been used in museums since at least the mid-twentieth-century (various U.S examples, including the Smithsonian) and later alternative dates for the uptick in the use of smell could be provided (such as the 1980s with Jorvik Viking Centre and other museums which used ambient scent as part of their curatorial strategy, inspired by themed attractions and in turn inspiring many others to follow). Either way, claims about what has or has not occurred over the past decade in particular need backing up with evidence. Have the authors compiled and tabulated a database of such examples or attempted to track this ostensible growth in the use of smell? I think the claim that we have witnessed something of a smelly renaissance is right (that’s certainly a vibe I can catch), but there’s a difference between feeling something is so and evidencing it.

 The second issue in the abstract, repeated in the main body of the text, is the argument that the deployment of scent in a museological, commercial, or educational context has had the aim of offering ‘visitors a direct sensory connection to the past; in other words, to collapse temporal distance, even if only momentarily, by allowing them to physically experience the air of a bygone era’. It is worth dissecting this claim in detail. The first point to make is that the authors have ascribed this aim to the users of smell but offered no evidence for that ascription. In this respect they share ground with Mark Smith, who has assumed that the goal of sensory reconstruction in a museum context is purely to beguile the audience into believing they are experiencing a form of time travel (see various publications of his, including most recently his *Sensory History Manifesto* and his co-authored book with Rob Boddice *Emotion, Sense, Experience*). However, no evidence is offered for this assertion about the intentions of those deploying scent or any indication that this is the way in which such deployments are experienced by audiences. In fact, some of the work on smell in a public history context has emphasised that smell *distances *us from the past rather than connecting us with it in a museum context (see Dolly Jorgenson, Louise D’Arcens). I am happy to share with the authors the accepted version of a paper due to be published in *The Public Historian *in August 2026 that deals with some of these issues should they wish to think them through further.

 More generally, there is a growing body of literature that deals with the question of reconstruction and the past. The much wider literature on historical dress, reconstruction of scientific experiments, or experimental archaeological reconstructions of food etc offers one literature. But there is also a small but evolving range of scholarship that explicitly discusses this in relation to the senses. Probably most obvious here is the literature on taste. One of the best recent examples comes from the thoughtful article co-authored by a group led by Marieke M. A. Hendriksen titled 'Tasting the Past? Developing a Methodology for Researching Historical Tastes'. This scholarship raises interesting questions about the distinction between material fidelity and the reconstruction of mere perception that are also at issue here. These could do with more engagement in the article.

 The third issue is one of terminology. The article claims that the various approaches to re-creating scents from the past are ‘often lumped together under the broad and vague label of “reconstitution”’. Again, a claim is made that something ‘often’ occurs and then offers no evidence of said occurrences.  Who is doing this lumping? The public? The media who report scientific findings? The scientific authors of papers? This is not clear. Perhaps this is because the assertion is false: to my knowledge I have not observed a tendency within the literature – or within a public facing museums and heritage context – to universally deploy the word ‘reconstitution’.  A variety of terms have been used – reconstruction, recreation, reinterpretation and so on.  In fact, the authors themselves describe endeavours that ‘re-create’, ‘reconstruct’, or use historical material to ‘inspire’.  Sure enough, in footnote 3 the article then goes on to admit that this is a term that the authors *themselves *have ‘chosen to use in this work to refer to all olfactory compositions claiming a historical dimension, regardless of the method used to create them’. So, in fact the authors are the ones doing the lumping together and not the projects and practitioners they are surveying. The paragraph in which the ‘lumping’ accusation occurs does include an example of sorts, referring to the deployment of reconstructions of ancient Egyptian or seventeenth-century French perfumes by museums without ‘providing any information on the sources consulted or the techniques employed’.  This is an important point. However, it is not clear why this particular problem is relevant to the objective of the article. There is a conversation to be had – in fact, one that is already being had - about how one uses and displays smells in the context of museums, but that is not the ostensible objective of this article. Such a discussion requires thoughtful engagement with questions in the museum studies literature around curation, labelling, audience experience, diffusion methods, etc (because otherwise it sounds like the solution offered by the authors of this paper is a big block of text setting out the method, materials, and aims of a reconstitution, which I’m pretty sure would be ineffective for a lot of exhausted museum goers!). This means that the reference to museum practice feels like a straw person or a distraction from the real issue of classifying different methods of reconstructing smells.

 That lumping together brings me to a fourth issue, which is the precise ontological status of ‘scent’ in this piece. In the introduction, for example, the stated aim is to develop a vocabulary for classifying attempts to bring ‘lost scents back to life’. As is clear and appropriate, the expertise of the Osmothéque concerns perfumes (we might use the word ‘scent’ here as an alternative way of describing intentionally designed olfactory compositions) but it has not to my knowledge (and I am happy to be corrected here) been interested in the evocation of smellscapes or non-perfumery-related smells. Now it is clear that the modern development of smells for use in a *museum*  setting (I am deliberately excluding living history and heritage sites here, which often unintentionally diffuse smells as result of the practices that occur within them) since the mid twentieth century has often had the effect of converting all attempts at evoking any smell whatsoever from the past (whether a perfume or an ambient smellscape) into *a scent* by virtue of the technologies and techniques of reconstruction, manufacture, and diffusion used in museums. The smell of hell (to take an example discussed in the article), that might have once been evoked by the stench of a leather-tanner’s pit in a medieval mystery play or the sulphuric squibs used to create sound effects on the early modern stage or evoked in the minds of worshippers by a preacher’s words is instead converted into a bottled artifact that can be spritzed and sprayed (though not too liberally). This produces a difficult tension for this classificatory system to resolve. For whilst most if not all attempts to evoke the smells of the past use the technologies of modern perfumery, only some and not all the scents thus revivified were modern perfumes to begin with. I think there needs to be more recognition in the article that some of the examples are perfumes that recreate perfumes but others are perfumes that smells or smellscapes. This basic distinction seems to really be at the heart of the classification thus enumerated (and makes we wonder whether really we are dealing with two classifications here: one for perfumes and another for smellscapes or ambient scents or whatever term you would like to use). At the moment the classification blurs the two and I am not sure if that really helps.

 I do like the idea of the graphical representations of different aspects of a particular reconstruction. However, I do feel for these to be comprehensible (and re-used in other articles for classifying other scents) more explanation of the 15 different criteria, how those criteria were chosen, how they are ‘rated’ (and who does the rating) etc. Could it be suggested, for example, that such graphical representations might be published with future scent reconstructions in other journals or part of the peer review process of a reconstructed scent (see the Odeuropa work in *American Historical Review *where a peer review of a smell was used, which was also used in the Coughlin et al’s recent publication of an article accompanied by scratch and sniff card). If so – and I think this could be a nice way to add to and develop the idea of peer reviewing reconstructions – it would behove the authors to develop a more concrete outline of the 15 criteria and how they are assessed. If these are to be used in the context of public engagement and mediation, then the public(s) thus engaged need to be made aware of the criteria and what they mean. Which 'public(s)' are these criteria aimed at engaging?

 A few other things I picked up on: I was confused by the statement in the abstract that the classification was based on ‘objective criteria and was independently conceived, without any evaluative or promotional intent’. I would have thought this goes without saying and the statement about ‘objective’ criteria probably raises the issue of subjectivity that is best left in its box. There also seemed to be some confusion about what constitutes 'the past'. In discussing the painting ‘Orange Blue’ (1964-5) by Ellsworth Kelly the authors suggest that though 'the artwork is actually modern, it was treated as a source from the past'. This sentence appears to operate on the basis of a conception of 'the past' that I am unfamiliar with. My understanding is that 1964-5, rather like the 25 ^th^ of December 2025, is very much in the past. At present their does, despite protestations to the contrary, appear to be a hierarchy at work – a hierarchy of mimesis. We start with the formulas, closest to the scents, and we end with works that might not even have any mimetic function at all and thus are judged to be outwith the boundaries of what is ‘historical’. All classifications such as this are boundary drawing exercises and all boundary drawing exercises serve different interests. I think we should not shy away from admitting this. I think I have offered enough reflection here and so I will avoid commenting on the afterword which, whilst interesting, felt like a separate article answering a separate important question (about *why *the fascination with historical reconstruction of past smells has ostensibly grown in significance over the last decade) that needed space for its own engagement with scholarship, evidence, and footnotes. There is much more I would love to write and engage with here at more length but I am afraid that the deadline for the review was very tight and sent just before Christmas. I have therefore had limited time to write this review.

If any, are all the source data and materials underlying the results available?

No source data required

Is the work original in terms of material and argument?

Yes

Is the work clearly and cogently presented?

Partly

Is the argument persuasive and supported by evidence?

Partly

Does the research article contribute to the cultural, historical, social understanding of the field?

Yes

Does it sufficiently engage with relevant methodologies and secondary literature on the topic?

Partly

Reviewer Expertise:

I am a historian of smell and a public historian interested in the role of the senses in our contemporary engagement with the past.

I confirm that I have read this submission and believe that I have an appropriate level of expertise to confirm that it is of an acceptable scientific standard, however I have significant reservations, as outlined above.

# Response

**Published:** 2026-04-27

**Authors:** Isabelle CHAZOT

**Affiliations:** Laboratoire de Recherche Historique Rhone-Alpes, Lyon, Auvergne-Rhône-Alpes, France

We would like to thank Dr. Tullett for his evaluation, insightful comments, and suggestions aimed at improving the manuscript. We have taken most of his remarks into account and have revised the article accordingly. His feedback has helped us clarify several conceptual and methodological aspects of the NOMEN framework. We respond below to the main issues raised.

**1. Clarification regarding the “Whiffstory Toolkit”** We thank the reviewer for pointing out the inaccuracy in our reference to a “Whiffstory Toolkit.”  We have revised the manuscript to refer accurately to the relevant publications produced within the Odeuropa project, including Whiffstory: Using Multidisciplinary Methods to Represent the Olfactory Past and Making Whiffstory, and we have removed the incorrect reference. This gave us the opportunity to clarify the scope of application of the NOMEN framework. In particular, we emphasized the model’s specificity in that it includes perfumers’ expertise in the olfactory interpretation of sources and focuses on the creation of odorous compositions.

**2. Framing of the field and claims about recent proliferation** Our reference to “the past decade” and our characterisation of a recent proliferation of historically inspired scent projects did not intend that the use of scent in museum did not exist before (we agree that the use of scent in museums and heritage contexts has a longer history, including significant developments in the mid- to late 20th century), but that it recently became more important. Indeed this assertion was not based on statistics, but on our collective observations and on bibliometry – this recent surge is also noted in the article mentioned above (Whiffstory... “In the last years, we have witnessed a growing trend toward employing scent in a heritage context.” p. 862). Rather than situating the phenomenon exclusively within the last decade, we have decided to make clear that those initiatives were already existing in the past, but with less visibility.  Our aim was not to deny earlier precedents, but to highlight the increasing density and methodological diversity of contemporary practices. We have moderated the formulation accordingly.

**3. Attribution of intentions (“collapsing temporal distance”)** We acknowledge that a formulation suggesting that these projects aim to “collapse temporal distance.” may risk attributing a singular intention to a heterogeneous range of initiatives. We have revised this passage to emphasise that historically inspired olfactory projects are presented with different goals, and that their relationship to temporal mediation is neither uniform nor unproblematic. Rather than asserting that they “collapse temporal distance”, we now describe them as offering forms of sensory engagement with historical contexts, the effects of which depend on both curatorial framing and audience reception. We acknowledge that “smells/scents” can create a distance from the past rather than connecting us to it

**4. Terminology and the question of “lumping together”** We have clarified that NOMEN itself constitutes an analytical effort to distinguish between practices that are often referred to under umbrella terms, whether in institutional communication, media discourse, or professional usage. We had already specified that the term “reconstitution” was used to give a name to those many endeavours, whatever term was used originally. In fact, the mis-use of “reconstitution”, “reconstruction” or “recreation” is less relevant  for our perspective than the absence of description in the method employed.

**5. Ontological status of “scent”: perfume / smellscape** In response to the observation regarding the ontological tension between historically attested perfumes and smellscapes or ambiant odours, we now state more explicitly that, although NOMEN emerged from expertise in perfumery, it applies to contemporary olfactory compositions that claim a historical dimension, whether these concern the recreation of a specific perfume or the evocation of a historical environment. We consider that these objects are not ontologically equivalent and that their inclusion within a shared classificatory framework reflects a common contemporary practice of mobilising the past through scent technologies. As Dr Tullett writes himself in his comment “all attempts to evoke the smells of the past use the technologies of modern perfumery”.   Moreover, the Osmothèque has begun to incorporate into its collections olfactory compositions with historical aims, such as those produced within the context of Odeuropa

**6. Adaptation and reconstruction: methodological boundaries** We had already stressed that boundaries between categories were not always clear and couldn’t be perfectly impermeable. We agree that adaptation and reconstruction may overlap in practice, especially where chemical analysis and textual sources intersect. We have clarified that NOMEN assigns categories based on the primary methodological claim guiding the composition rather than on the exclusive use of a single type of source. We have also refined the discussion of “reconstruction” to distinguish between cases involving residual olfactory continuity and those based solely on inferred molecular evidence. Our aim is not to deny hybridity, but to provide a framework capable of describing dominant methodological orientations while acknowledging overlaps.

**7. Graphic representation and the fifteen criteria** We appreciate the reviewer’s interest in the radar charts and his suggestion that greater transparency regarding the criteria and their assessment would strengthen their  effectiveness as a mediation tool. We have clarified that the scoring system is heuristic rather than metric, and that the values attributed reflect structured qualitative assessments based on available documentation. We emphasise that the purpose of the diagrams is comparative and descriptive, not evaluative. While we have not expanded the framework into a fully standardised protocol, we have added clarification regarding the interpretative dimension involved in the attribution of values.

**8. Hierarchy and the notion of “the past”** It was not our intention to establish a hierarchy of mimetic legitimacy, but rather to organise the categories along a spectrum of source directness and creative latitude. We have clarified that this continuum is analytical rather than normative. Regarding the example of Ellsworth Kelly’s Orange Blue, we have revised the phrasing to avoid suggesting that the 1960s fall outside “the past.” Our intention was to refer to the use of a temporally prior artwork as a historical source within the framework of the project, not to imply a restrictive definition of historicity.
